# HIV-1 Vpu interacts with RBM10 to promote HIV-1 infection

**DOI:** 10.1128/msystems.00403-25

**Published:** 2025-07-31

**Authors:** Boye Li, Yanzhe Hao, Xianbin Meng, Xiaoming Wang, Qingyan Li, Runqing Jia, Yishu Yang, Jinning Yang, Boyang Yu, Tian Chen, Wenmei Zhang, Xiaoguang Zhang, Xiayan Wang, Qin Hu

**Affiliations:** 1 College of Chemistry and Life Science, Beijing University of Technology 12496, Beijing, China; 2 Civil Aviation Medicine Center, Civil Aviation Administration of China 91582, Beijing, China; 3 National Key Laboratory of Intelligent Tracking and Forecasting for Infectious Diseases, National Institute for Viral Disease Control and Prevention, Chinese Center for Disease Control and Prevention 198050, , Beijing, China; 4 MOE Key Laboratory of Bioinformatics, Center for Synthetic and Systematic Biology, School of Life Sciences, Tsinghua University 12442, , Beijing, China; 5 The Key Laboratory of Geriatrics, Beijing Institute of Geriatrics, Institute of Geriatric Medicine, Chinese Academy of Medical Science, Beijing Hospital/National Center of Gerontology of National Health Commission 56688 , Beijing, China; 6 Beijing International Science and Technology Cooperation Base of Antivirus Drug, Beijing University of Technology 12496, Beijing, China; 7 Center of Excellence for Environmental Safety and Biological Effects, Beijing Key Laboratory for Green Catalysis and Separation, Beijing University of Technology 12496, , Beijing, China; National Institutes of Health, National Institute of Allergy and Infectious Diseases, Bethesda, Maryland, USA

**Keywords:** viral protein U, RNA-binding motif protein 10, engineered ascorbate peroxidase labeling, protein-protein interaction

## Abstract

Viral protein U (Vpu), an accessory protein of HIV-1, functions by antagonizing or hijacking various host factors to allow the virus to evade host immune surveillance and facilitate viral release. Nevertheless, there is limited understanding regarding the impact of Vpu on the biogenesis of HIV-1 RNAs. In this study, we utilized ascorbate peroxidase 2 (APEX2)-based proximity labeling techniques in combination with mass spectrometry and immunoprecipitation-mass spectrometry (IP-MS) to characterize interactions between HIV-1 Vpu and host proteins. We identified nine cellular targets of Vpu. Among these targets, our research demonstrated the interaction between Vpu and RNA-binding motif protein 10 (RBM10), which results in the degradation of RBM10 through the ubiquitin-proteasome pathway. The expression of RBM10 exerted an inhibitory effect on virus replication by binding to viral RNA and reducing the levels of incompletely spliced HIV-1 transcripts. Additionally, it promoted the transcription of various antiviral genes. The findings elucidate the role of HIV-1 Vpu in RNA replication and identify RBM10 as a novel regulator of HIV-1 transcription.

**IMPORTANCE:**

A comprehensive analysis utilizing APEX2-MS and IP-MS techniques identified a total of 24 cellular targets of Vpu, three of which have been documented as restriction factors. Vpu-interacting proteins were found to be significantly enriched in pathways related to cell adhesion, RNA transport, and the spliceosome. The identification of RBM10 as a novel regulator of HIV-1 replication and infectivity, and RBM10 regulated transcription of both viral and host RNA transcripts. Vpu interacted with RBM10 and promoted its degradation through the ubiquitin-proteasome pathway.

## INTRODUCTION

Human immunodeficiency virus type 1 (HIV-1), the pathogenic retrovirus responsible for acquired immunodeficiency syndrome (AIDS), continues to pose a significant global public health challenge. According to the World Health Organization (WHO), estimated 39.9 million individuals worldwide were living with HIV by the end of 2024 (1). The pathogenesis of HIV-1 infection encompasses complex interactions between viral replication and host factors, which serve as critical determinants of disease outcome. Elucidating these intricate virus-host interactions is essential for both understanding HIV-1 pathogenesis and developing next-generation therapeutic strategies. Nevertheless, the nature of these interactions is still not well understood.

Accumulating evidence has substantiated the critical role of HIV accessory proteins in virus-host interactions, particularly in the regulation of viral infectivity and host immune response (2). Viral protein U (Vpu) is an 81-residue accessory protein that is unique to HIV-1 and has homologous variants in specific strains of simian immunodeficiency virus (SIV). Although not critical for viral replication, Vpu is integral to the later stages of the viral lifecycle and is essential for the propagation of HIV-1. It facilitates virus release and aids in evading immune surveillance. Multiple studies have demonstrated that Vpu functions as a multifunctional protein, antagonizing various host restriction factors (3, 4). By far, bone marrow stromal cell antigen 2 (BST-2) and CD4 are two significant targets of Vpu (5, 6). BST-2, commonly known as tetherin, is a broad-spectrum viral restriction factor that inhibits the release of various enveloped viruses by tethering them to the cell membrane (7). Vpu interacts with BST-2 via its transmembrane domain and post-translationally counteracts the activity of BST-2 by diminishing its presence on the cell surface, hindering its circulation, and facilitating intracellular degradation (3, 8–10). Furthermore, Vpu impedes CD4 within the endoplasmic reticulum by inducing Beta-transducin repeats-containing proteins (β-TrCP)-mediated ubiquitination and subsequent degradation through the endoplasmic reticulum-associated protein degradation pathway (6). The downregulation of cellular BST-2 and CD4 enhances viral release and provides protection to the infected cells against antibody-dependent cell-mediated cytotoxicity (8, 11). Additionally, Vpu also modulates the expression of various host factors, including human leukocyte antigen C (HLA-C) (4, 12), NK-T-B antigen (NTB-A) (13), CD1d (14), intercellular adhesion molecule 1 (ICAM-1) (15), poliovirus receptor (PVR) (16), CD47 (17), and T cell immunoglobulin and mucin domain-containing protein 3 (Tim-3) (18). A recent study has identified a novel effect of Vpu on the accumulation of newly produced viral cDNAs (19). Existing evidence suggests that Vpu not only targets host restriction factors but also impacts viral replication processes. However, the mechanism by which Vpu impacts viral replication remains to be elucidated.

Proteomics has emerged as a pivotal platform for elucidating the intricate interaction between the host and the virus in HIV-1 pathogenesis. Sugden et al. (15) and Matheson et al. (20) conducted Stable isotope labeling with amino acids in cell culture (SILAC)-MS analysis on the plasma membrane proteome of T cells expressing Vpu, leading to the identification of the cell adhesion molecule intercellular cell adhesion molecule-1 (ICAM-1) and the amino acid transporter Sodium Coupled Neutral Amino Acid Transporter 1 (SNAT1) as HIV-1 restriction factors that are downregulated by Vpu. Additionally, Liu et al. (21) employed an isobaric tag for relative and absolute quantitation (iTRAQ)-MS to analyze HIV-1-infected primary CD4 T cells and identified P-selectin glycoprotein ligand-1 (PSGL-1) as an HIV-1 restriction factor which is counteracted by Vpu. In this study, we utilized a proximity-labeling technique in conjunction with mass spectrometry (APEX2-MS) to examine the repertoire of cellular proteins that interact with Vpu in TZM-bl cells. A comparable methodology has been effectively employed by another research group (22). Through this methodology, we successfully quantified a total of 136 proteins, 9 potential cellular candidates subsequently authenticated through immunoprecipitation MS orthogonal validation. Notably, we found a new RBM10-Vpu interaction. RBM10 is an RNA-binding protein implicated in talipes equinovarus, atrial septal defect, Robin sequence, and persistent left superior vena cava (TARP) syndrome and various cancer types (23, 24). Recent research has identified the antiviral role of RBM10 in dengue virus infection, which is mediated by the splicing of the antiviral protein spermidine/spermine acetyltransferase 1 (SAT1) and the activation of various pro-inflammatory cytokines (25). However, the role of RBM10 in the biogenesis of HIV-1 infection remains unexplored. Our research identifies RBM10 as a novel regulator of HIV-1 viral replication. Additionally, it demonstrates that RBM10 directly interacts with viral RNA and regulates both viral transcripts and host antiviral genes. Moreover, Vpu antagonizes RBM10 by facilitating its degradation via ubiquitination.

## MATERIALS AND METHODS

### Cell culture and transfection

HEK293T, Jurkat, and HeLa cell lines were acquired from the Chinese National Infrastructure of Cell Line Resources (NICR, China). TZM-bl cells were acquired from the National Institutes of Health AIDS Research and Reference Reagent Program. The TZM-bl (JC, 53bl-13) cell line is a genetically modified variant of the HeLa cell line, which expresses the CD4 receptor as well as the chemokine receptors CCR5 and CXCR4. TZM-bl cells contain a coding sequence for firefly luciferase that is regulated by the HIV-1 Tat-responsive long terminal repeat promoter. These cells are frequently utilized for the evaluation of viral infectivity. HEK293T, TZM-bl, and HeLa cells were cultured in Dulbecco’s modified Eagle’s medium (Gibco, NY, USA) supplemented with 10% fetal bovine serum (FBS) (Gibco, NY, USA) and penicillin-streptomycin (Gibco, NY, USA). Jurkat cells were cultured in RPMI 1640 medium (Gibco, NY, USA) supplemented with 15% fetal bovine serum and penicillin-streptomycin. HEK293T, HeLa, and TZM-bl cells were transfected utilizing Lipofectamine 3000 Transfection Reagent (Invitrogen, CA, USA) following the manufacturer’s instructions. For the construction of RBM10-overexpressing Jurkat T cell lines, the cloning vectors for lentivirus were developed by inserting *RBM10* genes into the pLenti-CMV-GFP-ccdB-puro vector (Tsingke Biotech, China) at the NheI and AscI restriction sites, and the empty pLenti-CMV-GFP-ccdB-puro vector serves as a control. HEK293T cells were co-transfected with pLenti-CMV-GFP-ccdB-puro vector or the empty pLenti-CMV-GFP-ccdB-puro vector and packaging plasmids to produce infectious particles. The harvested lentivirus (MOI = 20) was used to infect Jurkat T cells in the presence of polybrene at 6 µg/mL, followed by 3–4 days of culture. Stable RBM10-overexpressing Jurkat T cells and control Jurkat T cells were generated through puromycin selection.

### Plasmids and small interfering RNAs

The HIV envelope (Env) expression plasmid pREJO4541.67 (NIH#11035) and the backbone vector pSG3^∆env^ (NIH#11051; NIH AIDS Research and Reference Reagent Program) were obtained from the National Institutes of Health AIDS Research and Reference Reagent Program. The *Apex2* and *RBM10* genes were synthesized by Tsingke Biotech (China). Vectors containing APEX2-Vpu and APEX2-NES (nuclear export sequence) fusions were generated by inserting the fused genes into the pcDNA3.1 vector at the BamHI and EcoRI restriction sites, and the empty pcDNA3.1 serves as a control. All the siRNAs were purchased from KeyGEN (China). The sequences of the siRNAs used in this study are as follows: hRBM10 si-1 sense, CUUCGCCUUCGUCGAGUUUAGTT; hRBM10 si-1 antisense, CUAAACUCGACGAAGGCGAAGTT.

### *In situ* APEX-2-mediated proximity labeling

Proximity labeling using APEX-2 was performed as previously described (26). HeLa cells were transfected with either pcDNA3.1-Vpu-APEX2 or pcDNA3.1-APEX2-NES. Twenty-four hours post-transfection, the cells were incubated with 500 mM biotinyl tyramide (Sigma, MO, USA) for 30 min, followed by the addition of H_2_O_2_ (final concentration of 1 mM) for 1 min at room temperature to initiate the biotinylation process. The reaction was then quenched in a quencher solution (5 mM Trolox, 10 mM ascorbic acid, and 10 mM sodium azide in PBS) for 30 s. After the final quenching, the cells were collected and lysed in RIPA buffer (Solarbio, China) containing a protease inhibitor cocktail (Promega, WI, USA) on ice. The lysates were subjected to centrifugation at 12,000 × *g* for 15 min at 4℃, and the supernatants were collected.

### Streptavidin-based affinity purification of biotinylated proteins and on-bead proteolytic digestion

Following the assessment of protein concentration utilizing the Pierce 660 nm protein assay (Thermo Fisher Scientific, MA, USA), the supernatant was incubated with streptavidin magnetic beads overnight at 4℃. The beads were washed twice with RIPA lysis buffer, once with 1 M KCl, once with 0.1 M sodium carbonate, once with 2 M urea (dissolved in 10 mM Tris-HCl, pH 8.0), and twice with RIPA lysis buffer. Alexa Fluor 568-conjugated streptavidin (Thermo Fisher Scientific, MA, USA) was subsequently employed to monitor protein biotinylation. For on-bead digestion, beads were washed twice with 50 mM Tris-HCl buffer (pH 7.5) and twice with 2 M urea/50 mM Tris buffer (pH 7.5), pre-digested for 1 h at room temperature with trypsin in 2 M urea/50 mM Tris buffer containing 1 mM dithiothreitol (DTT), and again washed twice with 2 M urea/50 mM Tris buffer. The eluted supernatant was treated with 4 mM DTT for 30 min at room temperature. Following the reduction process, the sample was subjected to alkylation with 10 mM iodoacetamide for 45 min and subsequently digested overnight at room temperature in a trypsin solution.

### Proteomic analysis

Peptide samples were analyzed using a Thermo Orbitrap Fusion Mass Spectrometer in conjunction with a Thermo-Dionex Ultimate 3000 High-Performance Liquid Chromatography system. Peptides were resolved on a homemade fused silica capillary analytical column (Upchurch, WA, USA) packed with C-18 resin (Varian, MA, USA) at a flow rate of 0.300 µL/min. The mobile phase A was composed of 0.1% formic acid, while mobile phase B comprised 100% acetonitrile and 0.1% formic acid. Mass spectrometry analysis was conducted in data-dependent acquisition mode with full scans (350–1,550 *m*/*z*) acquired using an Orbitrap mass analyzer at a mass resolution of 120,000. Tandem mass spectrometry (MS/MS) scans were conducted for 3 s using higher-energy collisional dissociation (HCD), with the HCD energy parameter established at 30%. The data were collected utilizing Xcalibur 3.0 software, and the proteomics data were analyzed employing the Proteome Discoverer application (version 2.4) (Thermo Fisher Scientific, MA, USA). The CV values among biological replicates were performed online in BioLadder (bioladder.cn). After data preprocessing including missing value imputation and normalization, *t*-tests were conducted to calculate the fold change and p-value for each protein. Proteins meeting the criteria of log_2_fold change (Log_2_FC) > 2 and *P* < 0.05 were subsequently subjected to Gene Ontology (GO) and Kyoto Encyclopedia of Genes and Genomes (KEGG) pathway enrichment analyses using R (R Foundation for Statistical Computing, Austria).

### Western blot and antibodies

HeLa cells were lysed on ice for 15 min in RIPA lysis buffer (Solarbio, China) supplemented with a protease inhibitor cocktail (Promega, WI, USA). Following the determination of protein concentration using a bicinchoninic acid protein assay kit (Solarbio, China), the protein samples were resuspended in NuPAGE LDS sample buffer, which included NuPAGE reducing reagent (Invitrogen, CA, USA) and subsequently heated at 95℃ for 5 min. The proteins were then separated on 12% Mini-PROTEAN TGX gels (Bio-Rad, CA, USA), transferred onto polyvinylidene difluoride membranes, blocked with 5% nonfat milk in PBS containing 0.01% Tween 20, and then incubated first with anti-V5 antibody (Thermo Fisher Scientific, MA, USA), anti-human RBM10 antibody (Abcam, UK), or anti-human β-actin antibody (Abcam, UK) overnight at 4℃ and then with horseradish peroxidase-labeled goat anti-rabbit secondary antibody (Cell Signaling Technology, BSN, USA) or goat anti-mouse secondary antibodies (Cell Signaling Technology, BSN, USA). Proteins were identified utilizing an enhanced chemiluminescence system (Beyotime Biotechnology, China). The protein bands were visualized utilizing a Tanon 5200 multi-imaging system (Tanon Science & Technology, China). For the degradation pathway assay, HeLa cells expressing Vpu-APEX2 were pretreated for 6 h with brefeldin A (Selleck Chemicals, TX, USA; BFA, 200 nM; ER-Golgi transport inhibitor), MG132 (Selleck Chemicals, TX, USA; 20 µM; proteasome inhibitor), 3-MA (Selleck Chemicals, TX, USA; 10 mM; autophagy inhibitor), or niclosamide (Selleck Chemicals, TX, USA; 50 µM; DNA replication inhibitor) before the end of the 48 h culture period. RBM10 expression was then assessed by western blot.

### Co-immunoprecipitation and co-IP–MS

HeLa cells were transfected with either pcDNA3.1-Vpu-APEX2 or pcDNA3.1-APEX2-NES. After 48 h, the cells were subjected to three washes with phosphate-buffered saline (PBS) and subsequently lysed using Pierce IP Lysis Buffer (Thermo Fisher Scientific, MA, USA), which was supplemented with protease inhibitors (Promega, WI, USA). The lysate was incubated with anti-V5-tag monoclonal antibodies conjugated to magnetic beads (MBL, China) for 2 h at 4℃. The beads were washed four times with a cold washing buffer composed of 50 mM Tris-HCl (pH 7.5), 150 mM NaCl, and 0.05% NP-40. Subsequently, the protein was eluted by boiling for 5 min in NuPAGE LDS sample buffer containing NuPAGE sample reducing reagent. The supernatant was collected and used for SDS-PAGE and western blot analysis using an anti-human RBM10 antibody. In the context of co-immunoprecipitation coupled with mass spectrometry (coIP–MS), the proteins were separated on SDS-polyacrylamide gels and digested with trypsin before mass spectrometric analysis. The raw data were collected utilizing Xcalibur 3.0 software and processed using the Proteome Discoverer application (version 2.4). Proteins that interact with RBM10 were screened based on a standard of Log_2_FC ＞ 2, score ＞ 3.

Reciprocal co-immunoprecipitation was performed as follows: cells were collected, washed three times with ice-cold PBS, and lysed in 1 mL RIPA buffer containing protease inhibitors, followed by pre-incubation with protein A/G agarose beads (Beaver, Wuhan) at 4℃ for 30 min. Lysates were equally divided into Input, IP, and IgG control groups and then incubated overnight at 4℃ with rotation using 2 µg anti-RBM10 polyclonal antibody (Proteintech, CN) and IgG antibody (Cell Signaling Technology, BSN, USA), respectively. After three washes with ice-cold buffer, antigen-antibody complexes were eluted in washing buffer followed by the addition of loading buffer, 5 min boiling, and centrifugation to collect supernatants. PAGE Electrophoresis was conducted on 4%–12% Bis-Tris gels (200 V constant) before silver staining (Solarbio, China) using the following protocol: The PAGE gel was fixed at room temperature for 30 min. The gel was then washed three times with deionized water and transferred to a silver staining solution for 40 min with gentle agitation. After staining, the gel was developed for 10 min at room temperature and stopped with a stop solution. The anti-human β-actin antibody (Abcam, UK) or anti-V5 antibody (Thermo Fisher Scientific, MA, USA) was used for the western blot detection.

### Preparation of viruses and infectivity analysis

For virus release detection, HEK293T cells were transfected with the pcDNA3.1-RBM10 plasmid, empty pcDNA3.1 control plasmid, RBM10 siRNA, or control siRNA. Four hours post-transfection, the cells were co-transfected with the HIV envelope (Env) expression plasmid pREJO4541.67 (NIH#11035) and the pSG3^∆env^ backbone vector (NIH#11051; NIH AIDS Research and Reference Reagent Program). After 2 days, the Env-pseudotyped virus was harvested from the supernatants, and its infectivity was assessed utilizing the TZM-bl assay, as documented in prior studies (27, 28). In summary, TZM-bl cells were cultured in a 96-well plate and subsequently infected with the virus (1,000 TCID_50_/mL) diluted in a medium containing 10 µg/mL DEAE-dextran (Sigma, MO, USA). Two days post-infection, the infectivity of the viruses was quantitated by p24 enzyme-linked immunosorbent assay (ELISA) with the HIV-1 p24/Capsid Protein p24 ELISA Kit (Sino Biological, China) and assessed via luciferase assay employing the Bright-Glo Luciferase Assay System (Promega, USA) (29, 30). To detect the effect of RBM10 on viral infection, RBM10 overexpression TZM-bl cells and RBM10 knocking down TZM-bl cells were infected with HIV-1 delta-Env-pseudotyped virus for 48 h, and the luciferase activity was measured in RBM10 overexpression and knocking down TZM-bl cells. Jurkat cells exhibiting elevated levels of RBM10 expression and control Jurkat cells were infected with HIV-1 NL4-3 at a MOI of 0.05 and subsequently incubated at 37℃. After 2 h, the cells were washed three times with PBS for 5 min each and finally suspended in 96-well plates at a density of 50,000 cells per well and cultured for 7 days.

For proteomics analysis, RBM10 overexpressing TZM-bl cells and control TZM-bl cells were infected with HIV-1 NL4-3 for 5 days. Proteins were extracted and separated, followed by digestion with trypsin for mass spectrometric analysis. Proteins meeting the criteria of Log_2_FC > 2 and *P* < 0.05 were subsequently subjected to GO pathway enrichment analyses using R.

### Immunofluorescence

HeLa cells were transfected with either pcDNA3.1-Vpu-APEX2 or pcDNA3.1-APEX2-NES. Twenty-four hours post-transfection, the cells were fixed in 4% paraformaldehyde and permeabilized with 0.5% Triton X-100. After blocking with normal goat serum (Solarbio, China), the cells were stained with rabbit anti-human RBM10 antibody for 2 h at 37℃ and then incubated with Alexa Fluor 594-conjugated goat anti-rabbit secondary antibody (Earthox, CA, USA) or Alexa Fluor 488-conjugated goat anti-mouse secondary antibody (Earthox, CA, USA). The cells were subsequently counterstained with DAPI (Solarbio, China) for 10 min at room temperature and were imaged using a confocal laser scanning microscope (FV1000, Olympus, Japan or LSM980, Zeiss, Germany). The Pearson correlation coefficient was calculated using the Image Pro Plus (Media Cybernetics, USA).

### Immunoprecipitation of ribonucleoprotein complexes

The RIP (RNA Immunoprecipitation) assay was performed using the Magna RIP RNA-Binding Protein Immunoprecipitation Kit (Millipore Corporation, MA, USA). TZM-bl cells were subjected to infection with the Env-pseudotyped virus. After 48 h, the cells were washed twice with ice-cold PBS, pelleted by centrifugation at 1,500 revolutions per minute (rpm) for 5 min at 4℃, and incubated in radioimmunoprecipitation assay (RIPA) lysis buffer supplemented with a protease inhibitor cocktail and an RNase inhibitor on ice for 5 min. After a 30 min pre-incubation of Protein A/G Magnetic Beads with either the anti-human RBM10 antibody or a control IgG antibody, the samples were subsequently incubated with the magnetic beads for 4 h and were washed five times using the RIP wash buffer. For the analysis of RNA immunoprecipitation, the beads were resuspended in a proteinase K buffer (RIP wash buffer with 10% SDS and proteinase K), and RNA was purified using an RNeasy Mini Kit (Qiagen, Germany). RBM10-bound RNA fragments were identified using the One Step TB Green PrimeScript PLUS RT-PCR Kit (Takara, Japan). The calculation of fold enrichment was conducted as follows:


(1) Fold enrichment=CT (output) / CT (input)

### Real-time PCR and reverse transcription PCR

Total RNA was extracted from the cells utilizing the RNeasy Mini Kit (QIAGEN, Germany). The relative expression levels of RBM10 and viral RNA were determined using the comparative Ct method and a One Step TB Green PrimeScript PLUS RT-PCR Kit (Takara, Japan) on the ViiA 7 Real-Time PCR System (Applied Biosystems, TX, USA). The splicing products were analyzed using reverse transcription PCR as previously described (31). The PCR products were separated using a 6% acrylamide-urea electrophoresis gel (Solarbio, China) and imaged using the iBright imaging system (Thermo Fisher Scientific, MA, USA). The sequences of the primers utilized in this study are listed in Table S1.

### NanoBiT assay

For the NanoBiT assay, the cloning vectors utilized for the LgBiT and SmBiT fusion proteins were supplied as part of the Nano-Glo Live Cell Assay System (Promega, Madison, WI, USA). The *Vpu* and *RBM10* genes were subcloned into the pBiT1.1-C [TK/LgBiT], pBiT2.1-C [TK/SmBiT], pBiT1.1-N [TK/LgBiT], and pBiT2.1-N [TK/SmBiT] vectors at the *XhoI* and *NheI restriction* sites, respectively. HEK293T cells were co-transfected with plasmids encoding Vpu and RBM10 at a ratio of 1:1. The empty vector was used as a negative control. At 48 h post-transfection, Nano-Glo Live Cell Substrate (Promega, WI, USA) was added, and luminescence was quantified utilizing an EnSpire Multilabel Plate Reader 2300 (PerkinElmer, MA, USA).

### Diaminobenzidine staining and electron microscopy

DAB staining and electron microscopy were conducted following the methods previously described (32). HEK293T cells were transfected with either pcDNA3.1-Vpu-APEX2 or pcDNA3.1-APEX2-NES. The cells were harvested and subsequently fixed in a 2% (wt/vol) glutaraldehyde solution (Electron Microscopy Sciences) within a sodium cacodylate buffer (composed of 100 mM sodium cacodylate and 2 mM CaCl_2_, pH 7.4) while maintained on ice for 60 min. The samples were subsequently rinsed three times in the same buffer and then treated for 5 min in a buffer containing 20 mM glycine to quench glutaraldehyde. This was followed by three 1 min washes in sodium cacodylate buffer. DAB-H_2_O_2_ buffer, consisting of 100 mM sodium cacodylate, 2 mM CaCl_2_, 0.5 mg/mL DAB, and 10 mM H_2_O_2_ was subsequently applied to the cells for 15 min, and the reaction was then terminated by the addition of 1% osmium oxide. The cells underwent five washes with water. The processes of resin embedding and sectioning were performed as previously described (33). Images were acquired utilizing a FEI TF20 transmission electron microscope (FEI, OR, USA).

### Statistical analysis

The data were analyzed utilizing GraphPad Prism 8 software (GraphPad Software Inc.) and are presented as means ± standard deviations. Differences among groups were evaluated using a one-way analysis of variance and were considered significant at *P* < 0.05.

The mass spectrometric data were analyzed utilizing Microsoft Excel 2016 and R Studio (version 4.0.3). The significance of the proteomics data was assessed by the Student’s *t*-test, and variance was assessed by an *F* test to ensure the appropriate statistical assumptions were used. Gene Ontology (GO) and Kyoto Encyclopedia of Genes and Genomes (KEGG) analyses were performed using the ClusterProfiler package in R.

## RESULTS

### Identification of the host targets of Vpu

**Fig 1:**
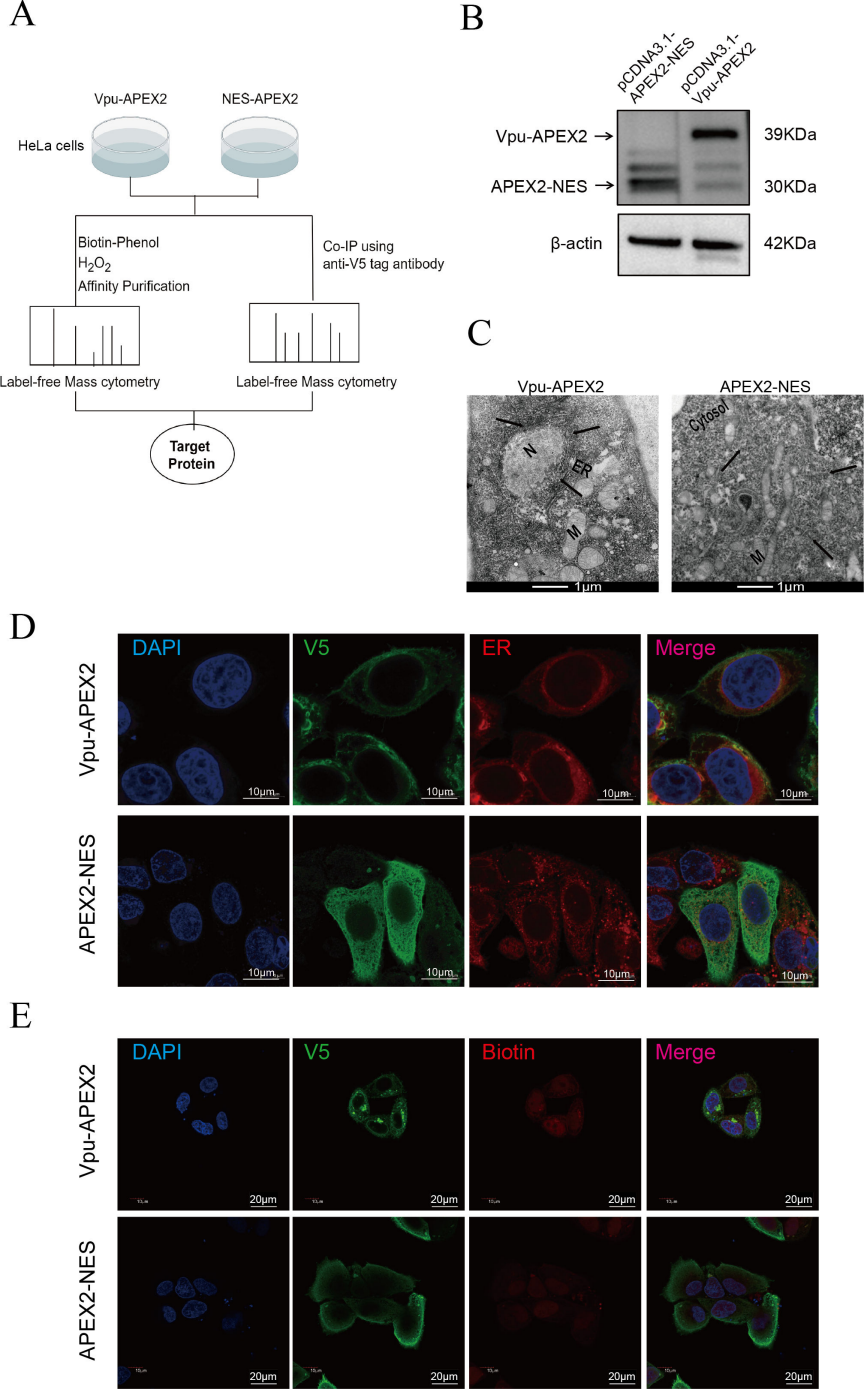
Intracellular localization of Vpu using the APEX2 proximity labeling analysis. (**A**) Schematic diagram of the APEX2 labeling. (**B**) HeLa cells were transfected with Vpu-V5-APEX2 or V5-APEX2-NES vector, respectively. After 2 days, the expression of the APEX2 fusion protein was assessed by western blot. (**C**) Cells expressing Vpu-APEX2 or APEX2-NES were incubated with 500 mM biotin tyramide for 30 min. After the addition of H_2_O_2_, the cells were stained with OsO4 and imaged using a transmission electron microscope. The fusion proteins of Vpu-APEX2 and APEX2-NES are indicated in the figure with black arrows. (**D**) HeLa cells were transfected with Vpu-V5-APEX2 or V5-APEX2-NES vector, the intracellular localization of Vpu was then determined by immunofluorescence. (**E**) HeLa cells were transfected with Vpu-V5-APEX2 or V5-APEX2-NES vector. After 2 days, the cells were incubated with 500 mM biotin tyramide for 30 min and then exposed to 1 mM H_2_O_2_ for 1 min. The expression of the APEX2 and biotinylated protein was evaluated by immunofluorescence. The data presented represent the pooled results from 2 to 3 independent experiments.

To investigate the interactions between Vpu and host proteins, APEX-proximity labeling and Co-immunoprecipitation coupled with mass spectrometry (coIP–MS) were applied (Fig. 1A). In brief, the *V5-Apex2* tag was fused to the *vpu* gene and cloned into the pcDNA3.1 vector, while the V5-APEX2-NES vector was utilized as a negative control. The expression of cellular Vpu-APEX2 and APEX2-NES was confirmed utilizing an anti-V5 antibody (Fig. 1B, duplicated results are shown in Fig. Fig. S1A and B). Additionally, the intracellular localization of Vpu was visualized through transmission electron microscopy (Fig. 1C, and duplicated results are shown in Fig. S1C through F) and immunofluorescence (Fig. 1D). The findings indicated that APEX2-NES was evenly distributed in the cytoplasm, while Vpu-APEX2 was mainly localized to the perinuclear region and various membranes, including endosomal and plasma membranes.

Upon the incubation of labeled cells with biotin-phenol and H_2_O_2_, APEX2 biotinylates neighboring proteins within a 20 nm radius of the fusion protein. Following the transfection of Vpu-APEX2, streptavidin blot analysis (Fig. S2A) demonstrated a substantial increase in the quantity of biotinylated protein in comparison to cells transfected with APEX2-NES. Furthermore, in accordance with cellular localization imaging analysis, biotinylated proteins that could potentially interact with Vpu were predominantly enriched in the endoplasmic reticulum and cell membrane, whereas potential APEX2-NES-interacting proteins showed diffuse cytosolic localization (Fig. 1E).

**Fig 2:**
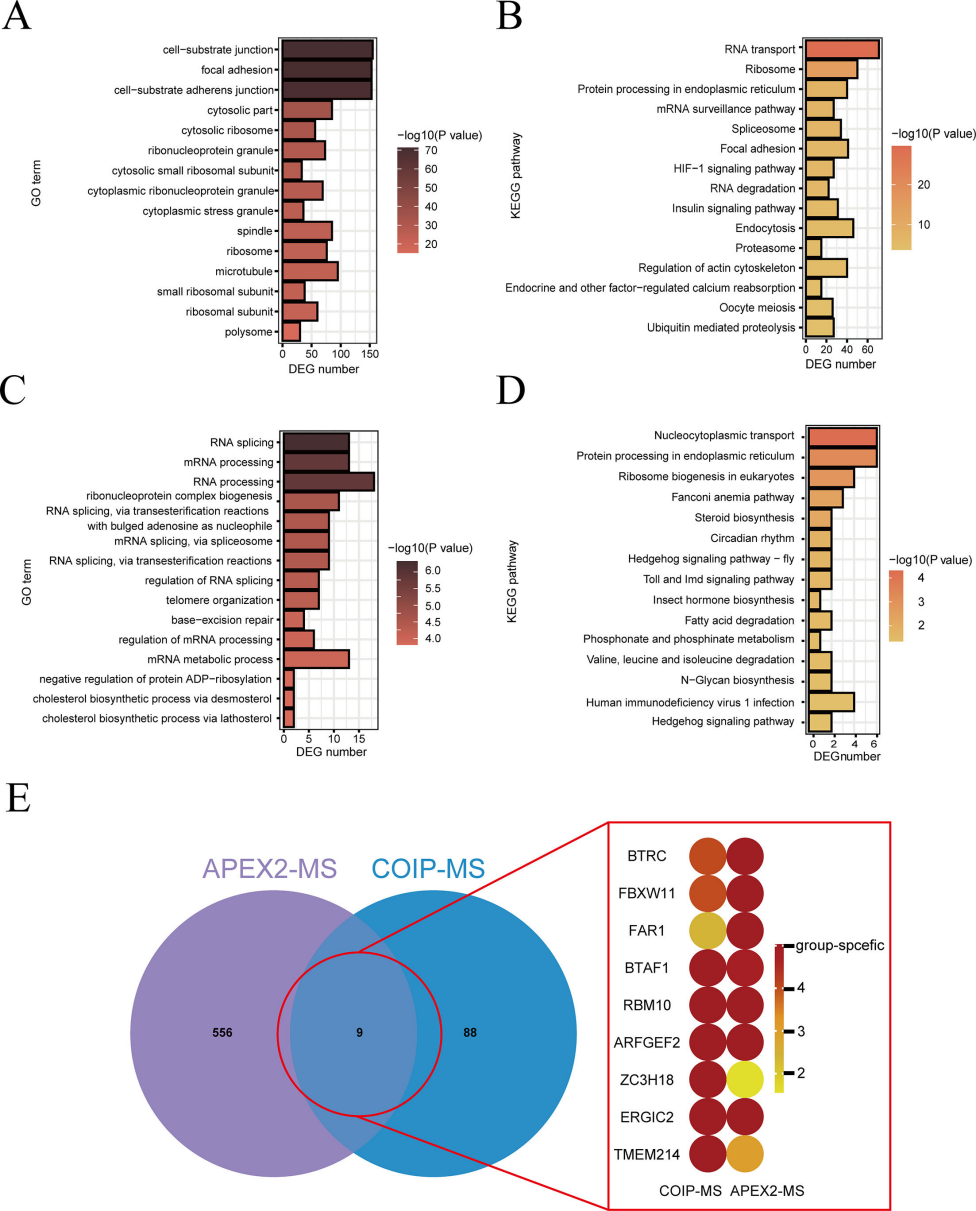
Proteomic analysis of Vpu–host protein interactions. After APEX2 proximity labeling, the biotinylated proteins were isolated and subjected to quantitative mass spectrometry. The GO (**A**) and KEGG (**B**) enrichment analyses were performed with 136 differentially expressed proteins (DEPs). Proteins were also enriched through co-immunoprecipitation–mass spectrometric (IP–MS), followed by GO (**C**) and KEGG (**D**) analysis. (**E**) The intersection analysis between APEX2-MS and IP-MS was performed and a total of 9 proteins were identified. The experiment was performed in triplicate and repeated 3 or 4 times.

Subsequently, we utilized streptavidin purification to enrich biotinylated proteins, which was followed by label-free quantitative (LFQ) mass cytometry. The APEX2-NES was employed as a control to eliminate interference from endogenous biotinylated protein (Fig. S2B). A total of 4,723 proteins were identified in this study, and the CV curve was shown in Fig. S2C. By applying the selection criteria of Log_2_FC > 2 and *P* < 0.05, we identified 136 proteins as potential targets of Vpu through a comparative analysis of enrichment between Vpu-APEX2 and APEX2-NES control groups (Table S2A). Furthermore, Gene Ontology (GO) analysis (Fig. 2A; Fig. S2D and E, Table S3B) and Kyoto Encyclopedia of Genes and Genomes (KEGG) (Fig. 2B, Table S3A) pathway enrichment analyses revealed that proteins interacting with Vpu were significantly enriched in pathways related to RNA synthesis, the spliceosome, RNA transport, and cell-substrate junctions.

To further validate the proteins that interact with Vpu, we performed a co-immunoprecipitation assay combined with mass spectrometry (IP–MS) using V5-tagged Vpu as the bait. Our research findings suggest that 97 proteins may interact with Vpu (Table S2B), and GO enrichment (Fig. 2C; Table S3D) and KEGG pathway (Fig. 2D; Table S3C) analysis indicated that these proteins were notably involved in pathways related to the regulation of RNA splicing, nucleocytoplasmic transport, and protein processing within the endoplasmic reticulum.

To ensure the identification of high-confidence protein targets, we performed an intersection analysis of the results obtained from APEX2- proximity labeling (Log_2_FC > 2, *P* < 0.05 or the protein uniquely detected in the Vpu group) and IP–MS analyzes (Log_2_FC > 2 and score ＞ 3). A total of 9 proteins were identified (Fig. 2E). Among the identified proteins, BTRC and FBXW11 are recognized as well-established targets that interact with Vpu (34, 35). Although the BST-2 known to interact with Vpu was not among the screened proteins mentioned above, it exhibited significantly high expression in both APEX2-proximity labeling (fold change = 1.221622631, *P* = 0.031) and IP–MS analyses (Vpu group-specific proteins) (36). In contrast, the other proteins have not been previously reported. Among them, RBM10 (an RNA-binding protein) regulates the alternative splicing of multiple host antiviral transcripts. Previous studies have implicated RBM10 in the pathogenesis of dengue virus and flavivirus (25, 37). An early genome-wide siRNA screen revealed that RBM10 siRNA inhibits VSV-G pseudotyped HIV-1 replication in 293T cells though the underlying mechanism remained unelucidated (38). To elucidate the function of Vpu in viral RNA replication, we selected RBM10 for further investigation.

### Vpu promotes RBM10 degradation through the ubiquitin-proteasome pathway

**Fig 3:**
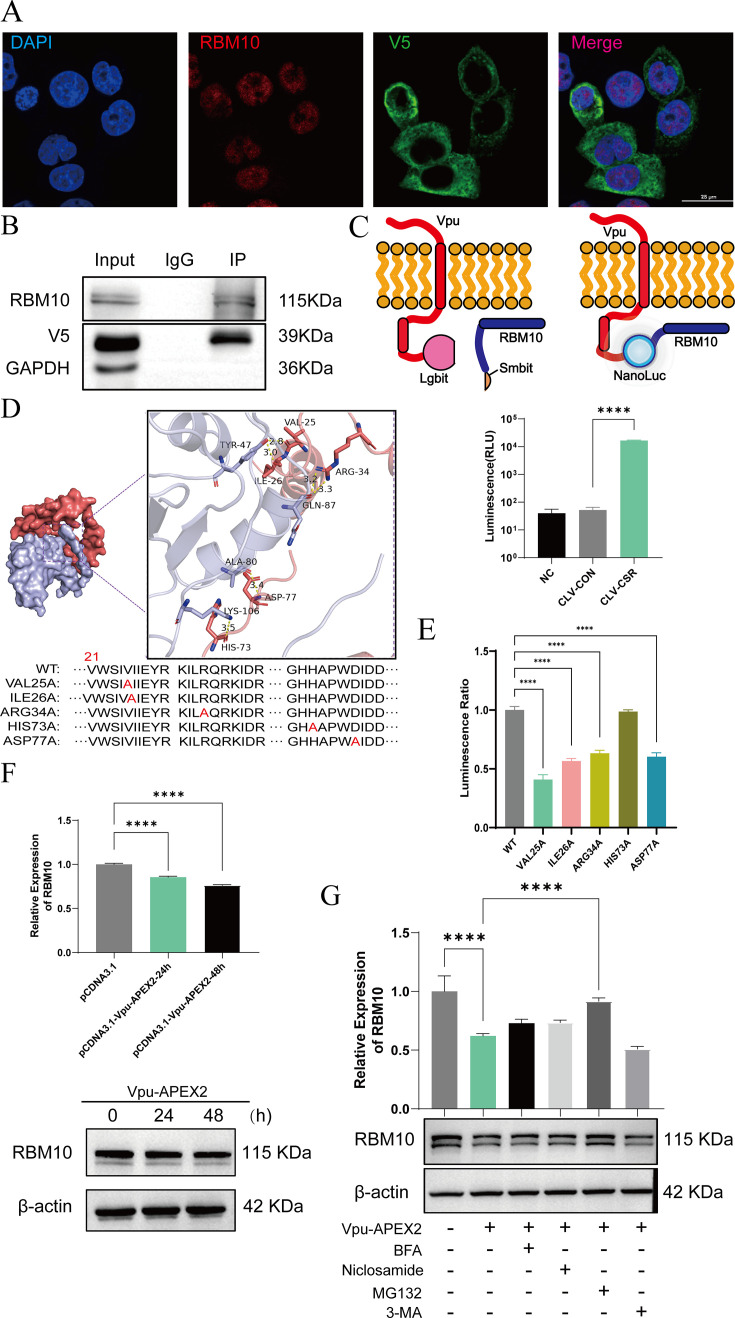
Vpu interacted with RBM10 and induced its degradation via the ubiquitin-proteasome pathway. (**A**) HeLa cells were transfected with the Vpu-V5-APEX2 vector, and the co-localization of RBM10 and Vpu was assessed by immunofluorescence. (**B**) Cell lysates were pre-incubated with Protein A/G beads and divided into Input, IP, and IgG groups. Lysates were incubated overnight with anti-RBM10 or IgG antibodies. The interaction between RBM10 and Vpu was measured by Co-IP analysis. (**C**) HEK293T cells were co-transfected with Vpu expression and RBM10 expression vector using the NanoBiT luciferase structural complementation reporter system. After 48 h, the luciferase signal was measured. The vectors used in experiments were CSR: pBiT2.1-C-rbm10 vector; CLV: pBiT1.1-C-vpu vector; NC: negative control; CON: SmBiT vector control. ****P < 0.0001 vs the NC group. (**D**) Docking analysis between Vpu and RBM10 proteins was performed using the docking server Hdock, and five point mutation vectors for Vpu were generated. (**E**) HEK293T cells were co-transfected with the RBM10 overexpression vector and point mutant vector of Vpu using the NanoBiT reporter system. After 2 days, the luciferase signal was quantified. ****P < 0.0001 vs the WT group. (**F**) HeLa cells were transfected with Vpu-APEX2 or the control vector, respectively. The expression of RBM10 was measured via western blot after 24 h and 48 h. ****P < 0.0001 vs the control group. (**G**) HeLa cells were transfected with Vpu-APEX2 vector for 48 h, Brefeldin A (BFA, 200 nM), MG132 (20 µM), 3-MA (10 mM), and niclosamide (50 µM) were added respectively 6 h before the end of the experiment. The expression of the RBM10 protein was measured by western blot. The values presented are the means ± standard deviation of three replicates. All the experiments were performed three times. ****P < 0.0001 vs the Vpu group.

To investigate the interaction between RBM10 and Vpu, a Co-IP assay was performed to confirm the co-immunoprecipitation of RBM10 with Vpu using either RBM10 (Fig. S3A; Fig. 3B, duplicated results are shown in Fig. S3B and C) or V5-tagged Vpu as the bait (Fig. S3D and E). Additionally, confocal microscopy was employed to observe the cellular localization of Vpu and RBM10. The results indicated that RBM10 co-localized with Vpu in the perinuclear region (Pearson coefficient: 0.403942; Fig. 3A). We subsequently employed a luciferase-based complementation reporter assay, known as NanoBiT, to analyze the binding interaction between Vpu and RBM10. The N-terminus and C-terminus of Vpu and RBM10 were fused with LgBiT and SmBiT, respectively. LgBiT and SmBiT are two split fragments of NanoLuc luciferase that can produce luciferase signals upon ligand-induced interactions. When LgBiT was fused to the C-terminus of Vpu and SmBiT was fused to the N-terminus of RBM10, the resulting construct produced a significantly higher luminescence signal compared to the control, indicating a direct protein-protein interaction between Vpu and RBM10 (Fig. 3C). Other fusion protein combinations exhibited a similar trend but with weaker luminescence signals (Fig. S3F).

To elucidate the binding sites between Vpu and RBM10, we utilized the SwissModel server to generate a 3D structure and conducted docking analysis utilizing HDOCK. Our findings indicated a significant binding affinity between RBM10 and Vpu, as demonstrated by a binding energy measurement of −219.78 kcal/mol (Fig. 3D). The docking analysis also predicted the formation of six hydrogen bonds between Vpu (residues HIS-73, ASP-77, ARG-34, ILE-26, and VAL-25) and RBM10 (residues LYS-106, ALA-80, GLN-87, and TYR-47). To further elucidate the specific amino acid residues implicated in this protein interaction, we constructed five NanoBiT luciferase reporter vectors, each incorporating a mutant binding site of Vpu. Our results demonstrated that mutations in VAL-25, ARG-34, ILE-26, and ASP-77 of Vpu protein significantly impacted the interaction between Vpu and RBM10 (Fig. 3E), whereas Vpu-BST-2 (tetherin) binding was significantly interfered by mutations in ASP-77, ARG-34 and VAL-25 (Fig. S4A). The data indicated that specific binding motif between Vpu and RBM10 via the transmembrane (TM) region of Vpu.

To further examine the effect of Vpu on the expression of endogenous RBM10, we conducted a western blot analysis to detect RBM10 expression levels 48 h following Vpu transfection. Our findings indicated that Vpu reduced the expression of RBM10 in a time-dependent manner (Fig. 3F, duplicated results are shown in Fig. S5A and B). Subsequently, we treated the cells with MG132 (proteasome inhibitor), BFA (protein transport inhibitor), niclosamide (DNA replication inhibitor), or 3-methyladenine (3-MA; autophagy inhibitor). The data showed that the expression of RBM10 was restored after 6 h of MG132 treatment (Fig. 3G, duplicated results are shown in Fig. S5C and D). However, the use of BFA, niclosamide, and 3-MA did not recover its expression. These findings suggested that Vpu induced the degradation of RBM10 via the ubiquitin-proteasome pathway.

### RBM10 inhibits HIV-1 infection

**Fig 4:**
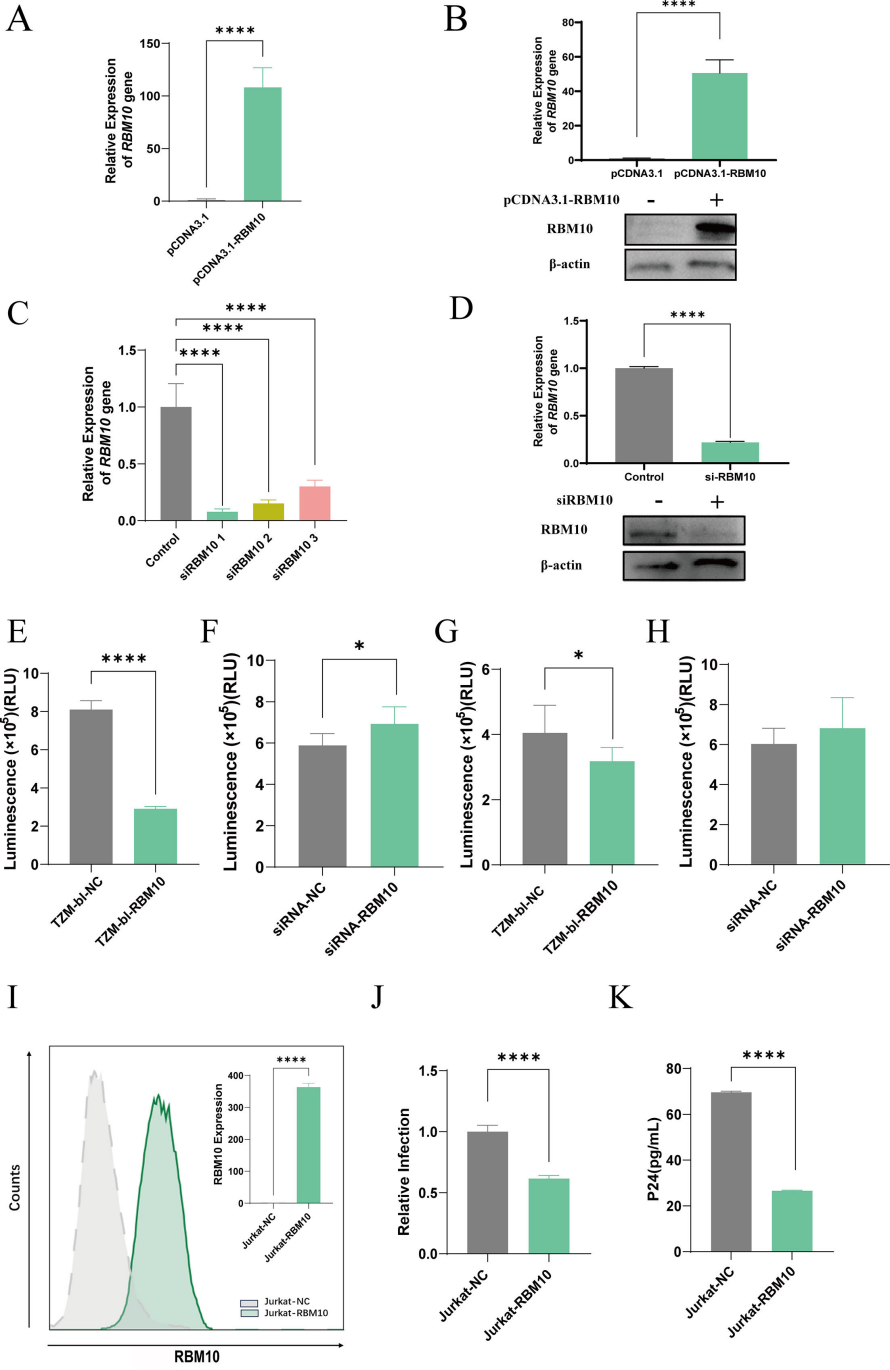
RBM10 regulated viral replication. TZM-bl cells were transfected with an RBM10 overexpression vector for 48 h. Western blot (**A**) and qPCR (**B**) were performed to confirm the overexpression of RBM10. TZM-bl cells were transfected with RBM10 siRNA for 48 h. Western blot (**C**) and qPCR (**D**) were performed to validate the efficacy of knocking down RBM10 in TZM-bl cells. Following transfection, cells were infected with HIV-1 delta-Env-pseudotyped virus for 48 h, and the luciferase activity was measured in RBM10 overexpression (**E**) and knocking down (**F**) cells. RBM10 overexpression HEK293T cells (**G**) and RBM10 knocking down TZM-bl cells (**H**) were co-transfected with proviral vectors; after 48 h, the supernatant was collected and the released virions were quantitated using the TZM-bl assay. (**I**) Jurkat cells were infected with RBM10 overexpression lentivirus or control lentivirus, and the expression of RBM10 was measured using flow cytometry analysis and RT-PCR. (**K**) RBM10 overexpressed Jurkat cells were infected with the HIV-1 NL4-3 virus (MOI = 0.05). After 7 days, the supernatant was collected, and the infectivity of the virus was measured using p24 ELISA (**J**) and TZM-bl assay. The values presented are the means ± standard deviation of three replicates. All the experiments were performed three times. *P < 0.05, **** *P* < 0.0001 vs the control group.

To investigate the function of RBM10 in viral infection, we conducted a series of infection assays. We established RBM10 overexpression (Fig. 4A and B; duplicated results are shown in Fig. S6A and B) and knockdown in TZM-bl cells (Fig. 4Cand D; duplicated results are shown in Fig. S6C and D), confirming that neither the overexpression nor the knockdown of RBM10 significantly affected the cellular activity of these cells (Fig. S7). Subsequently, we assessed viral infectivity using a *Luc* reporter assay conducted 48 h post-infection with Env-pseudotyped viruses. The finding indicated that RBM10 overexpression led to a reduction in viral infectivity (Fig. 4E), whereas the knockdown of RBM10 increased infectivity (Fig. 4F). Additionally, we transfected the cells with Vpu and its five mutants, which exhibited impaired binding activity with RBM10, respectively. The results indicated that Vpu mutants caused a reduction in viral infectivity when compared to the wild-type Vpu (Fig. S4B).

To further explore whether RBM10 impacts the production of new viral particles, we generated 293T cells with either RBM10 overexpression or knockdown, which were subsequently utilized for the packaging of Env-pseudotyped viruses. We evaluated the infectivity of these viral particles using the TZM-bl assay, revealing that RBM10 overexpression decreased the production of new infectious virions (Fig. 4G), whereas RBM10 knockdown appeared to enhance their production, although the differences were not statistically significant (Fig. 4H). Finally, we generated Jurkat T cells that overexpress RBM10 via lentiviral infection and subsequently infected these cells with the HIV-1 N4-3 virus (Fig. 4I). Viral infectivity was assessed using the TZM-bl assay (Fig. 4J) and anti-p24 capsid ELISA (Fig. 4K). The results indicated a significant reduction in viral replication in Jurkat T cells overexpressing RBM10. Collectively, these findings suggest that RBM10 exerts an inhibitory effect on HIV-1 viral infection.

### RBM10 directly binds to the viral RNA genome and regulates the transcription of both viral and host genes

**Fig 5:**
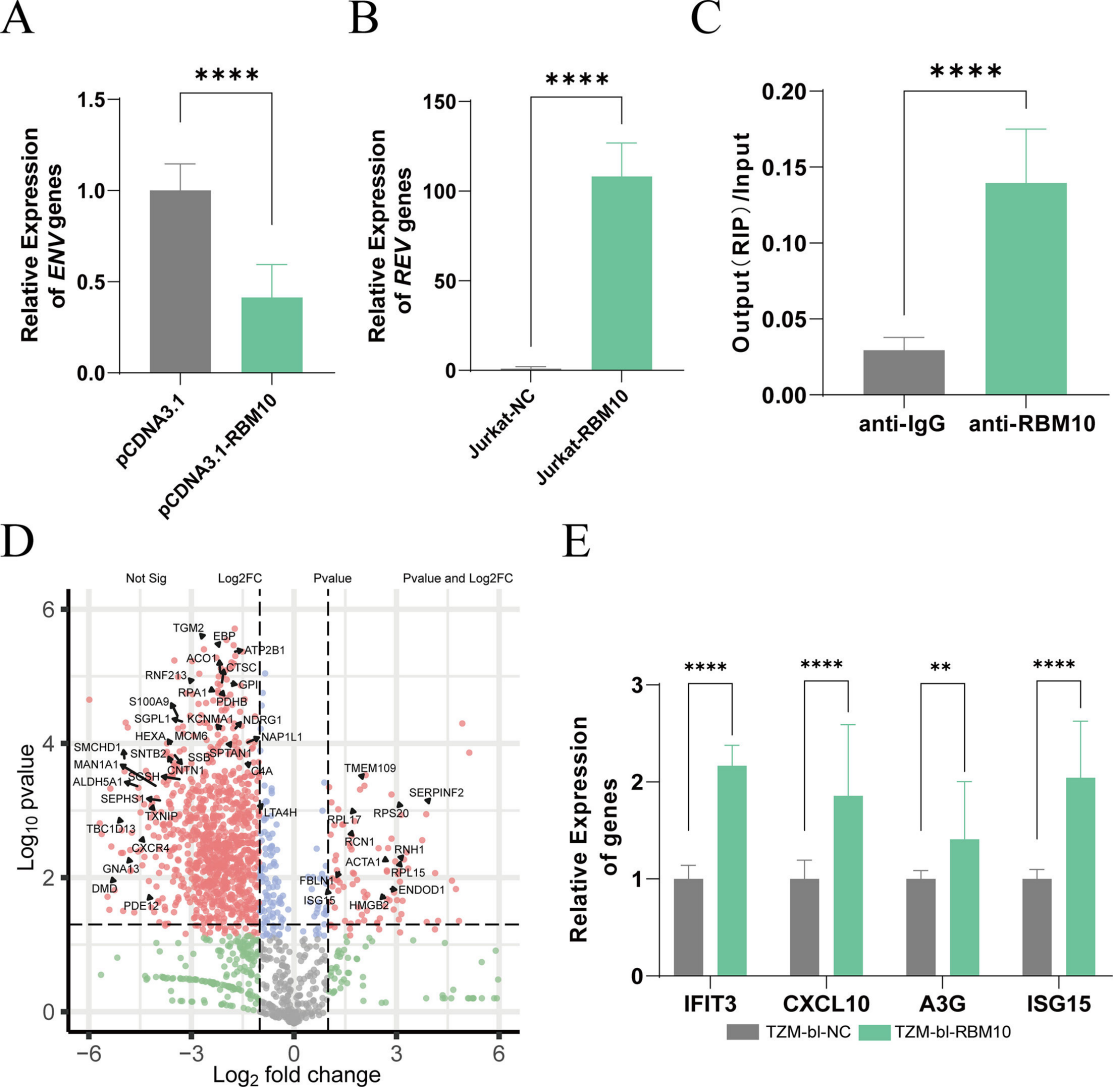
RBM10 regulated the expression of viral RNA transcripts and host antiviral factors. RBM10-overexpressing Jurkat cells and control Jurkat cells were infected with HIV-1 NL4-3 virus for 7 days. The virus RNA was extracted and detected by quantitative PCR (qPCR) using specific primers positioned on (**A**) Env (incompletely spliced transcripts) and (**B**) Rev (fully spliced transcripts). (**C**) RNA-immunoprecipitation (RIP) assays with anti-RBM10 of lysates from RBM10 overexpression TZM-bl cells. RT-qPCR was performed with primers of viral RNA and represented as output (RIP)/input RNA. (**D**) TZM-bl cells were transfected with either an RBM10 overexpression vector or a control vector for 48 h, followed by infection with the HIV-1 NL4-3 virus. After 5 days, the proteins were extracted from cells and subjected to LFQ proteomic analysis. A volcano plot was drawn by calculating the foldchange between the RBM10 overexpression group and the control group. (**E**) The transcription level of the antiviral factors IFIT3, CXCL10, APOBEC3G, and ISG15 were detected by real-time quantitative PCR. The values presented are the means ± standard deviation of three replicates. All the experiments were performed three times. **P < 0.01, ****P < 0.0001 vs the control group.

As RBM10 serves as a significant regulator of alternative splicing, we subsequently investigated its impact on viral RNA transcripts. We first infected Jurkat T cells that overexpress RBM10, as well as control T cells transfected with an empty lentiviral vector, with the HIV-1 NL4-3 virus. qPCR was employed to assess the gene expression levels of Env (Fig. 5A) and Rev (Fig. 5B), using total viral RNA (vRNA) as a loading control. The results indicated that the overexpression of RBM10 led to a reduction in the ratio of *env* transcripts to total viral RNA while increasing the ratio of *rev* transcripts to total viral RNA. Notably, Rev and Env are derived from distinct viral transcripts: Rev is encoded by the 1.8 kb fully spliced transcript, while Env is translated from the unspliced 4 kb transcript. To further elucidate these findings, we performed RT-PCR of 1.8 kb and 4 kb viral transcripts of TZM-bl cells infected with the HIV NL4-3 virus. Upon loading equal amounts of cDNA, we observed a decrease in the expression of the 4 kb intron-containing incompletely spliced transcript and an increase in the expression of the 1.8 kb intron-less completely spliced transcript (Fig. S8). Given that our study has found that RBM10 reduced the production of infectious viral particles, that data suggested that RBM10 inhibited the transcription of the 4 kb transcripts during the late stages of the viral life cycle. RNA immunoprecipitation (RIP) was subsequently performed on infected TZM-bl cells, demonstrating a significant enrichment of viral RNA when using the RBM10 antibody in comparison to the control IgG (Fig. 5C). The data suggested that RBM10 directly interacted with viral RNA to regulate its replication.

Subsequently, we performed label-free quantitative (LFQ) proteomic analysis on TZM-bl cells that were infected with the HIV-1 NL4-3 virus (Fig. S9A and B). A total of 4,443 proteins were identified in this study. Comparative proteomics analysis between the control and RBM10-overexpression groups revealed differential expression of 529 proteins, with criteria of Log_2_FC > 2 and *P* < 0.05 (Table S2C). The GO enrichment analyses indicated that RBM10 played a role in various viral processing pathways (Fig. S9C, Table S3E). A total of 44 proteins were identified as being involved in the viral process, including viral gene expression and the regulation of viral genome replication (GO:0016032, GO:0019058, GO:0019081). The data indicated that RBM10 is not only directly bound to HIV-1 RNA but also regulated factors that are involved in the transcription of HIV-1 RNA. Furthermore, among the differential expression proteins, we found 105 interferon-associated genes (IRGs) (21) that are often characterized as critical in the innate immune response against infectious diseases (Fig. 5D). We further validated the gene expression of four host antiviral factors in RBM10-overexpressing TZM-bl cells. As shown in Fig. 5E, the overexpression of RBM10 significantly increased the expression of the host restriction factors IFIT3, CXCL10, APOBEC3G, and ISG15. The data suggested that RBM10 regulated the expression of both viral transcripts and antiviral host factors.

## DISCUSSION

Mass spectrometry-based proteomics has become a powerful tool in clarifying novel host-virus interactions and the identification of new therapeutic targets (39–41). The APEX2 labeling method, known for its high sensitivity in live-cell proteomics, biotinylated adjacent proteins in a radius of 20 nm (26). Furthermore, the enzymatic activity of APEX2 enables its utilization in electron microscopy by catalyzing DAB. Using the APEX proximity labeling, Stoneham identified PTPN23 as a co-factor for HIV Vpu-induced BST-2 degradation (22). In the present study, we provide evidence for the efficacy of APEX2-MS in profiling transient Vpu-interacting proteins, and together with coIP-MS, we successfully identified nine potential cellular targets that exhibit binding affinity toward Vpu. Among the identified targets, we demonstrated the function of RBM10 in interfering with the transcription of viral RNA and cellular antiviral genes, consequently impeding HIV-1 replication. Furthermore, Vpu interacts directly with RBM10 and induces its degradation through the ubiquitin-proteasome pathway.

In the APEX2-MS assay, APEX2-NES was employed as the negative control to eliminate the potential influence of endogenous biotin interference. Through a comparative analysis of differential protein expression between APEX2-NES and Vpu-APEX2, we successfully identified 136 proteins that exhibited differential expression. These proteins were found to be enriched in cellular adhesion and proteasome pathways, which aligns with the findings reported by Stoneham et al. (22). Moreover, they also participated in RNA processing-related pathways, such as RNA transport and spliceosome. To validate and further refine the potential host targets of Vpu, we conducted co-immunoprecipitation followed by mass spectrometry analysis. Through intersection analysis, a total of nine proteins were identified. Among these, two proteins, namely BTRC and FBXW11(BTRC2), have already been established as cellular targets of Vpu (5, 34, 42). Upon binding to BST-2, Vpu recruits two F-box adapter proteins, BTRC and FBXW11 which are integral components of the SKP1/cullin1/F-box (SCF) E3 ubiquitin ligase complex. This recruitment initiates the polyubiquitination process of BST-2 (3, 43). Moreover, we discovered seven novel potential targets of Vpu, including RBM10, ARFGEF2, FAR1, and BTAF1. Vpu is a multifunctional accessory protein that plays a crucial role in facilitating virus release, mediating host-virus interactions, and evading the immune response (44, 45). In addition, recent work has revealed Vpu’s function in subverting DNA repair mechanisms to degrade nuclear viral cDNA in infected cells (19), indicating the role of Vpu in the transcription and replication of viral RNAs; however, little has been clarified yet. In light of the finding that Vpu participates in RNA processing, in our MS analysis, we selected RBM10, an RNA-binding protein as a target for further investigation.

RBM10 is widely expressed in nearly all human cells and more strongly expressed in actively transcribing cells. It regulates the alternative splicing of a large amount of target genes mostly by promoting the skipping of target exons from pre-mRNAs (46–49). In addition, RBM10 also regulated multiple RNA-binding events in a splicing-independent way (50). So, RBM10 was extensively involved in various physiological and pathological processes. Thus, investigating the interaction of Vpu and RBM10 will give a deeper understanding of Vpu’s role in viral replication and infection.

The interaction between Vpu and RBM10 was further validated through co-immunoprecipitation and immunofluorescence co-localization analysis. Previous research has demonstrated that Vpu primarily resides in the trans-Golgi network and the membranes of vesicles resembling multivesicular bodies (22, 51, 52), Volcic’s recent work has revealed that Vpu also localizes to the nuclear envelope/pores, enabling its interaction with nuclear pore proteins (19). Consistent with these findings, our study observed co-localization of Vpu and RBM10 in the perinuclear region of cells, suggesting that their interaction may occur in this specific cellular region. Furthermore, the NanoBiT complementation assay provided additional confirmation of their interaction. In this assay, NanoLuc luciferase is split into LgBiT and SmBiT fragments, which are fused to Vpu and RBM10, respectively. Upon interaction between the Vpu and RBM10 proteins, the two fragments approach each other closely, leading to the restoration of structure and enzymatic activity (53). Our findings indicate a significant signal following the binding of Vpu to RBM10. Additionally, when the N-terminus is occupied by the Nuc fragment, the signal weakens, suggesting that the binding primarily occurs in the N-terminus of Vpu. Furthermore, the homology modeling analysis indicates that the hydrogen bond between Vpu and RBM10 may involve HIS-73, ASP-77, ARG-34, ILE-26, and VAL-25 residues of Vpu, as well as LYS-106, ALA-80, GLN-87, and TYR-47 residues of RBM10. The data suggested that the TM region of Vpu and the N-terminal region of RBM10, which contain numerous modification sites (54), are implicated in this interaction. These findings were further supported by the observation that single point mutations in VAL-25, ARG-34, ILE-26, and ASP-77 of Vpu protein significantly attenuated the binding signal between Vpu and RBM10. Furthermore, Vpu was found to downregulate the expression of RBM10, which could be restored by treatment with MG132, indicating the involvement of the ubiquitin-proteasome pathway. In addition, 3-MA, an inhibitor of autophagy, was also assessed due to the involvement of a non-canonical autophagy pathway in the degradation of BST-2 induced by Vpu (55, 56). However, no significant recovery of RBM10 expression was observed, suggesting that Vpu-induced degradation of RBM10 occurs through a distinct pathway.

The impact of RBM10 on various cancer types and TARP syndrome has been extensively studied in recent years (23, 57, 58), and its role in viral infections is becoming increasingly evident. The involvement of RBM10 in viral infections is linked to its regulation of alternative splicing of host factors, such as SAT1 (25) as well as its interaction with lncRNA and the NF-κB signaling pathway (37). Notably, RBM10 has been shown to promote neuroinflammation during flavivirus infections while acting as a host restriction factor in dengue virus infections. The divergent functions of RBM10 prompted further investigations of its potential role in HIV-1 replication. Our findings demonstrated that the overexpression of RBM10 inhibits virus production, while RBM10 knockdown has the opposite effect, as observed using the Env-pseudotyped virus and NL-4.3 virus, indicating that RBM10 can serve as a novel host restriction factor of HIV-1 infection.

To explore whether RBM10 regulates viral RNA processing, we performed the RIP assay and found the direct binding of RBM10 to viral RNA. In the HIV-1 infection cycle, following viral integration, the approximately 9.7 kb proviral cDNA is transcribed into full-length pre-mRNA, which contains eight open reading frames (ORFs) and subsequently undergoes extensive alternative splicing. To date, the mechanisms underlying the alternative splicing of HIV RNA remain poorly understood, given that the single 9 kb HIV genome RNA can generate more than 50 viral transcripts. These transcripts can be classified into full-length 9 kb mRNA, 1.8 kb completely spliced mRNA, and 4 kb incompletely spliced mRNA. Generally, the shorter 1.8 kb intron-less mRNAs are spliced from the major splice donor D1 to the terminal acceptor A7, excising the central splice donor D4-A7 segment to encode Tat, Rev, and Nef. In contrast, the intron-containing 4 kb viral RNAs are spliced from D1 but retain the D4-A7 sequence with the RRE, allowing for the encoding of Vif, Vpu/Env, Vpr, and a one-exon-form of Tat transcripts. The unspliced 9 kb viral RNA, which encodes Gag and Gag-pol, also serves as genomic mRNA for packaging into new viral particles. In our study, we observed a significant reduction in the ratio of 4 kb mRNAs to total viral mRNAs in RBM10 overexpressed cells, implying that RBM10 may inhibit the transcription of intron-containing viral mRNAs. It is well established that during the early stages of infections, the proteins Tat, Rev, and Nef, encoded by the 1.8 kb intron-less mRNAs, are transported to the cytosol to facilitate the transition from early to late phases of infection, by enhancing transcription transactivation, nuclear export, and translation of the 4 kb and 9 kb mRNAs. In the late stages of the replication cycle, the 9 kb and 4 kb viral RNAs are transported to the cytoplasm in a Rev-dependent manner (59), where they encode the rest viral proteins, including the structural protein Env, Gag-pol, and accessory proteins Vpu, Vif, and Vpr, which are essential for the efficient viral replication, packaging, release, and infectivity (60). Since the 4 kb transcript encodes proteins including Env and Vpu, we propose that the Vpu protein, by degrading RBM10, can enhance the transcription of the 4 kb transcript and increase the translational expression of Env and Vpu proteins, thereby altering the transcriptional repression of the virus. Previous studies have shown that Vpu can promote the packaging of Env by degrading CD4 (61). Our data, along with previous studies, suggests that Vpu can enhance viral infection and release by promoting the expression and packaging of Env. Therefore, the alteration in the mRNA splicing dynamics induced by RBM10 may result in a reduction in the production of infectious new virions. RBM10 acts in the late stage of viral replication disrupts the balance of alternatively spliced viral mRNAs and reduces viral transcription.

Additionally, consistent with the previous findings indicating that RBM10 promotes the expression of TNF-α, IFN, and other pro-inflammatory genes, our results demonstrate an upregulation of several host antiviral genes in RBM10-overexpressing cells. These results suggest that RBM10 not only regulates viral RNA transcription but also participates in host gene expression. A number of RNA-binding proteins (RBPs), such as heterogeneous nuclear ribonucleoproteins (hnRNPs), including hnRNPs A0, hnRNP A1, and RBMX (62–64), have been reported to significantly affect the efficiency of viral replication. However, the precise mechanisms by which these proteins exert their effects, and the strategies used by the virus to counteract these host factors remain unresolved. Our findings identify RBM10 as a novel regulator in HIV-1 infection, affecting viral RNA transcription, and as a new target for Vpu in modulating viral replication. Given that current ART strategies can’t completely eradicate HIV-1 from the host, and prolonged treatment can induce drug-resistant mutations and various adverse effects, targeting HIV-1 alternative splicing sites may represent a promising strategy for therapeutic interventions (65). Several small-molecule compounds, including digoxin, chlorhexidine, and RNA interference (66–68), have been employed to suppress HIV-1 splicing and inhibit replication. Nevertheless, further investigation is necessary to comprehensively elucidate the mechanisms by which RBM10 affects viral RNA processes.

In this study, we employed the APEX2-MS technique in combination with coIP-MS to investigate the interaction between HIV-1 Vpu and host cell proteins. We identified a total of nine potential cellular targets. Notably, we discovered that the RBM10 protein directly binds to and reduces the levels of incompletely spliced viral RNA, while also enhancing the expression of various antiviral genes. Furthermore, Vpu interacts with RBM10 and initiates its degradation via the ubiquitin-proteasome pathway. Together, our findings reveal a novel role of Vpu and RBM10 in viral replication and provide a more comprehensive understanding of the interplay between the host and Vpu.

## Data Availability

The mass spectrometric data generated in this study have been submitted to ProteomeXchange (accession number: PXD039473).

## SUPPLEMENTAL MATERIAL

Supplemental Figures msystems.00403-25-s0001.docx Figures S1 to S9.

Table S1 msystems.00403-25-s0002.xlsx Primers used in this study.

Table S2 msystems.00403-25-s0003.xlsx Data set of candidate proteins.

Table S3 msystems.00403-25-s0004.xlsx Enriched pathways.

ASM does not own the copyrights to Supplemental Material that may be linked to, or accessed through, an article. The authors have granted ASM a non-exclusive, world-wide license to publish the Supplemental Material files. Please contact the corresponding author directly for reuse.
