# Supplementary material for: HIV-1 Vpu interacts with RBM10 to promote HIV-1 infection
Source: mSystems. 2025 Jul 31;10(8):e00403-25. doi: 10.1128/msystems.00403-25 (PMC12363165; doi:10.1128/msystems.00403-25)
Supplement: Supplemental Figures — Figures S1 to S9. [file msystems.00403-25-s0001.docx]

**
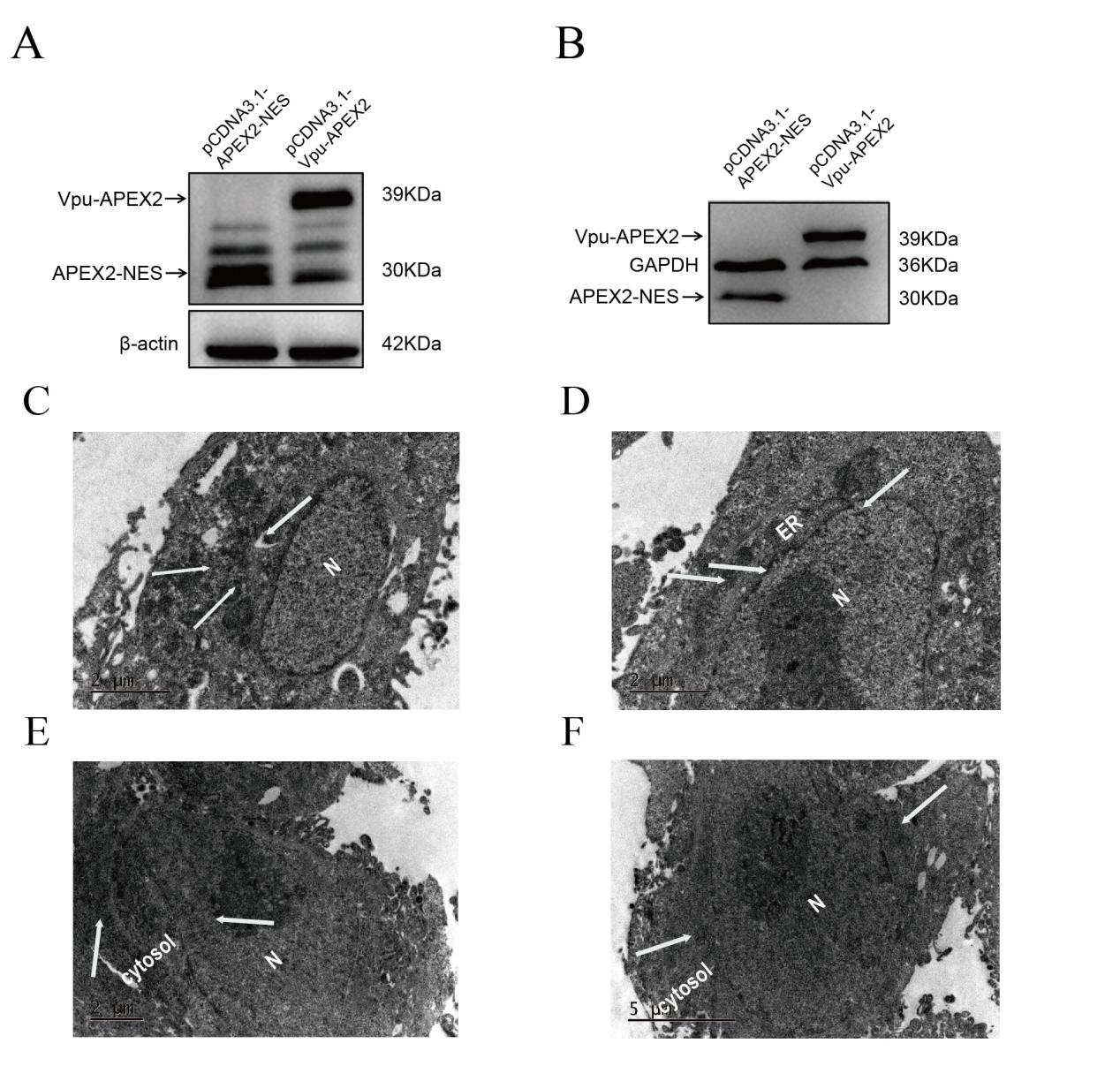
**

**FIG S1** (A-B) Replicate results of Fig 1B. HeLa cells were transfected with Vpu-V5-APEX2 or V5-APEX2-NES vector, respectively. After two days, the expression of the APEX2 fusion protein was assessed by western blot. (C-F) Replicate results of Fig 1C. The Hela Cells expressing Vpu-APEX2 or APEX2-NES were incubated with 500 mM biotin tyramide for 30 min. After the addition of H_2_O_2_, the cells were stained with OsO4 and imaged using a transmission electron microscope. The fusion proteins of Vpu-APEX2 and APEX2-NES are indicated in the figure with black arrows.


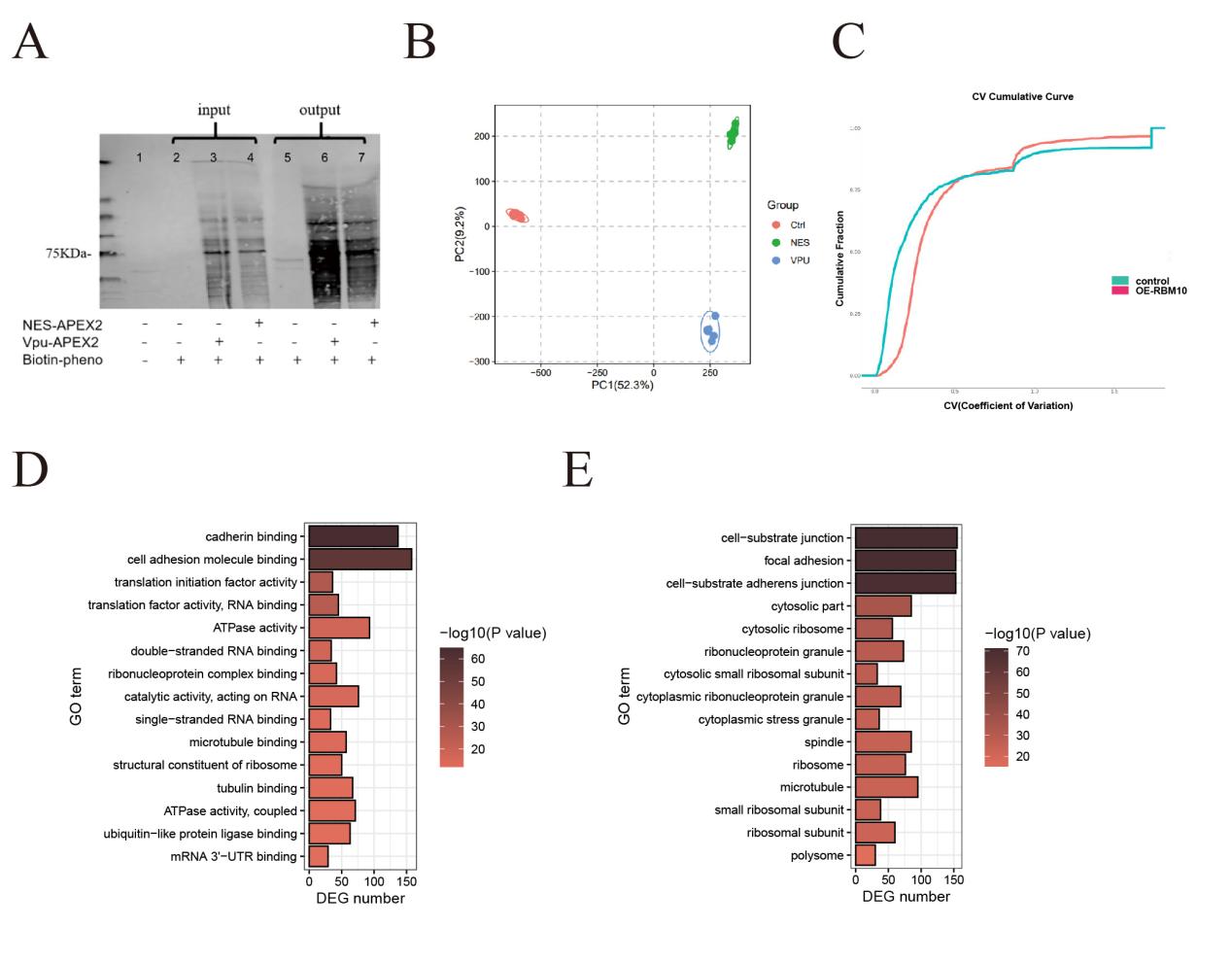


**FIG S2** Proteomic analysis of Vpu–host protein interactions.(A) HeLa cells were transfected with pcDNA3.1-Vpu-APEX2 or pcDNA3.1-APEX2-NES. HeLa cells were then incubated with 500 mM biotin tyramide, followed by the addition of H_2_O_2_ at a final concentration of 1 mM to initiate APEX2-mediated biotinylation. The biotinylated proteins were then enriched by streptavidin magnetic beads and subjected to SDS-PAGE analysis. The expression and enrichment of biotinylated proteins were detected using Alexa Fluor 568-conjugated streptavidin. (B) The enriched biotinylated proteins (representing Vpu-interacting proteins) from APEX2 proximity labeling were isolated and subjected to quantitative LC-MS. PCA analyses were performed to analyze the differences between groups. (C) The CV curve of APEX2-labeling proteomics. Enrichment analysis of differentially expressed proteins (DEPs) was performed using gene ontology (GO) mapping. (D) GO Molecular Function (MF) enrichment analysis. (E) GO Cellular Component (CC) enrichment analysis.


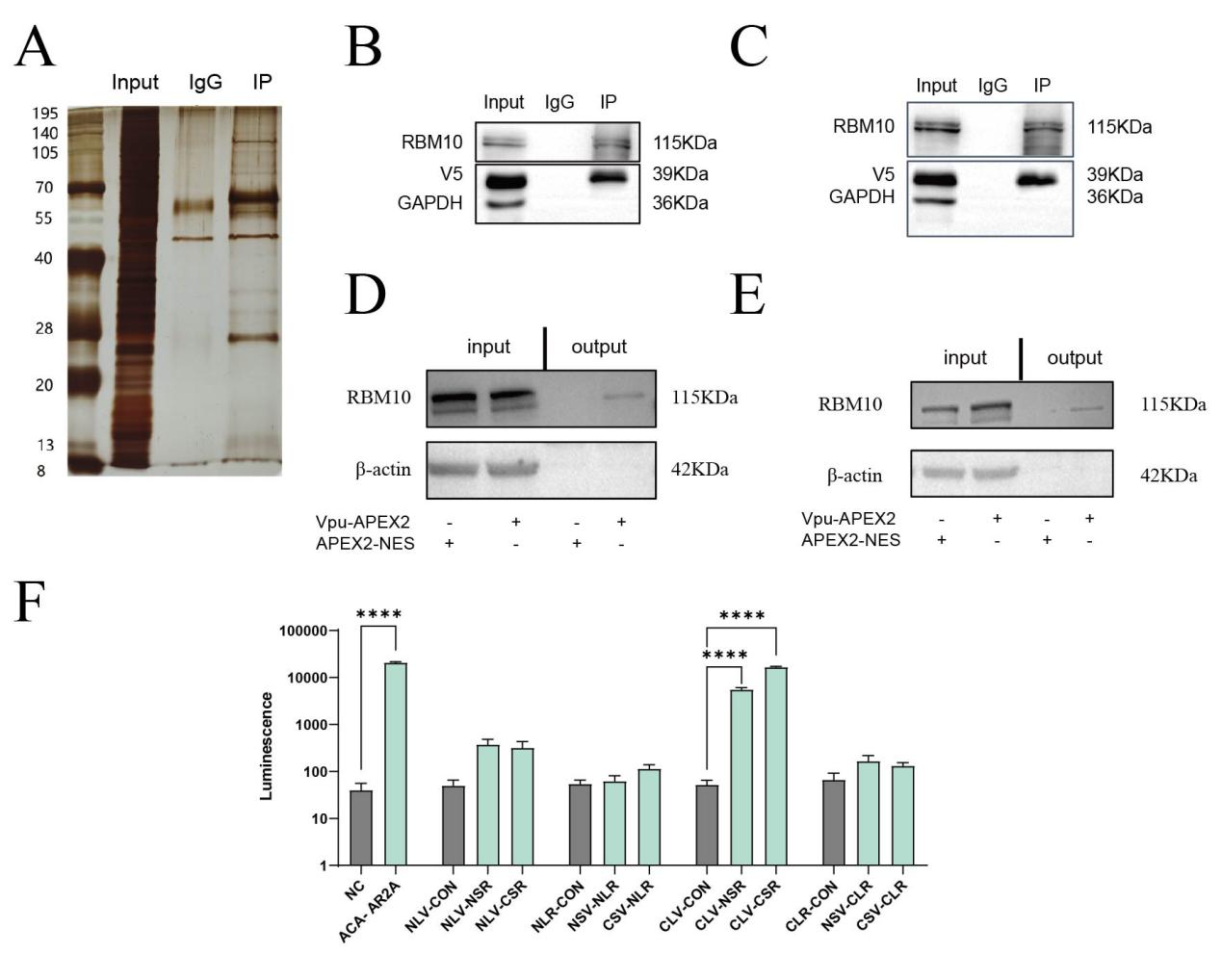


**FIG S3** (A) HeLa cells were transfected with the Vpu vector, and the silver staining experiment was performed to detect the enrichment of proteins enriched with RBM10 as bait. (B-C) Replicate results of Fig 3B. The interaction between RBM10 and Vpu was measured by Co-IP analysis. (D-E) The interaction between RBM10 and Vpu was measured by Co-IP analysis using V5-tagged Vpu as the bait. (F) HEK293T cells were co-transfected with Vpu expression and RBM10 expression vector using the NanoBiT luciferase structural complementation reporter system. After 48 h, the luciferase signal was measured. The vectors used in experiments were CSR:pBiT2.1-C-RBM10; CLR:pBiT1.1-C-RBM10; NSR:pBiT2.1-N-RBM10; NLR:pBiT1.1-N-RBM10; CSV: pBiT2.1-C-Vpu; CLV:pBiT1.1-C-Vpu; NSV:pBiT2.1-N-Vpu; NLV:pBiT1.1-N-Vpu; NC: negative control; CON: SmBiT vector control. ACA-AR2A: was used as a constitutive positive control. **** p < 0.0001 vs. the control group.


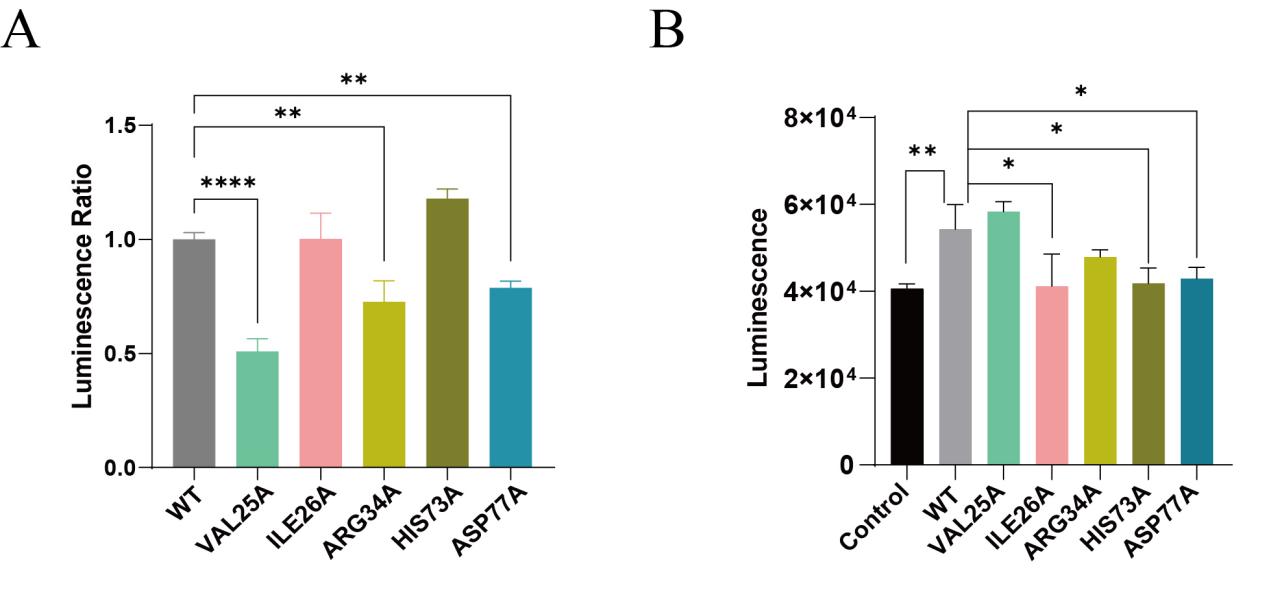


**FIG S4** (A) HEK293T cells were co-transfected with the BST-2 (Tetherin) overexpression vector and five point-mutant vectors of Vpu using the NanoBiT reporter system. After two days, the luciferase assays were performed to analyze the effect of mutation of the predicted binding site on the interaction between BST2 and Vpu. **p<0.01, **** p < 0.0001 vs. the *wt* control group. (B) The Tzm-bl cells transfected with Vpu and its five mutant vectors were infected with HIV-1 delta-Env-pseudotyped virus for 48 h, and the luciferase activity was measured to detect the viral infectivity.*p<0.05, **p<0.01 vs. the *wt* control group.


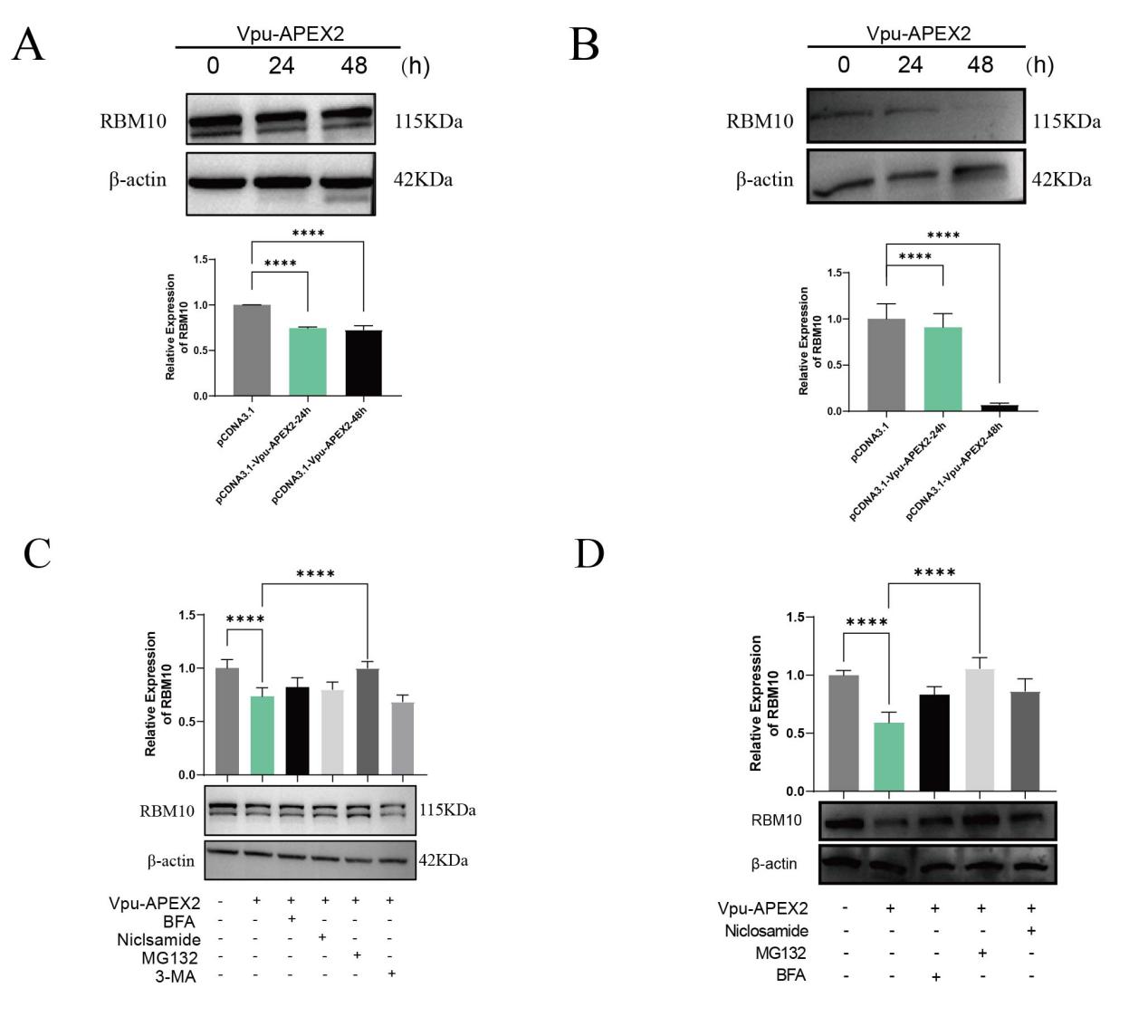


**FIG S5** (A-B) Replicate results of Fig 3F. HeLa cells were transfected with Vpu-APEX2 or the control vector, respectively. The expression of RBM10 was measured via western blot after 24 h and 48 h. (C-D) Replicate results of Fig 3G. Hela cells were transfected with Vpu-APEX2 vector for 48 h, Brefeldin A (BFA, 200 nM), MG132 (20 μM) 3-MA (10 mM), and niclosamide (50 μM) were added respectively 6 h before the end of the experiment. The expression of the RBM10 protein was measured by western blot. The values presented are the means ± standard deviation of three replicates. All the experiments were performed three times. **** p < 0.0001 vs. the control group or Vpu group.


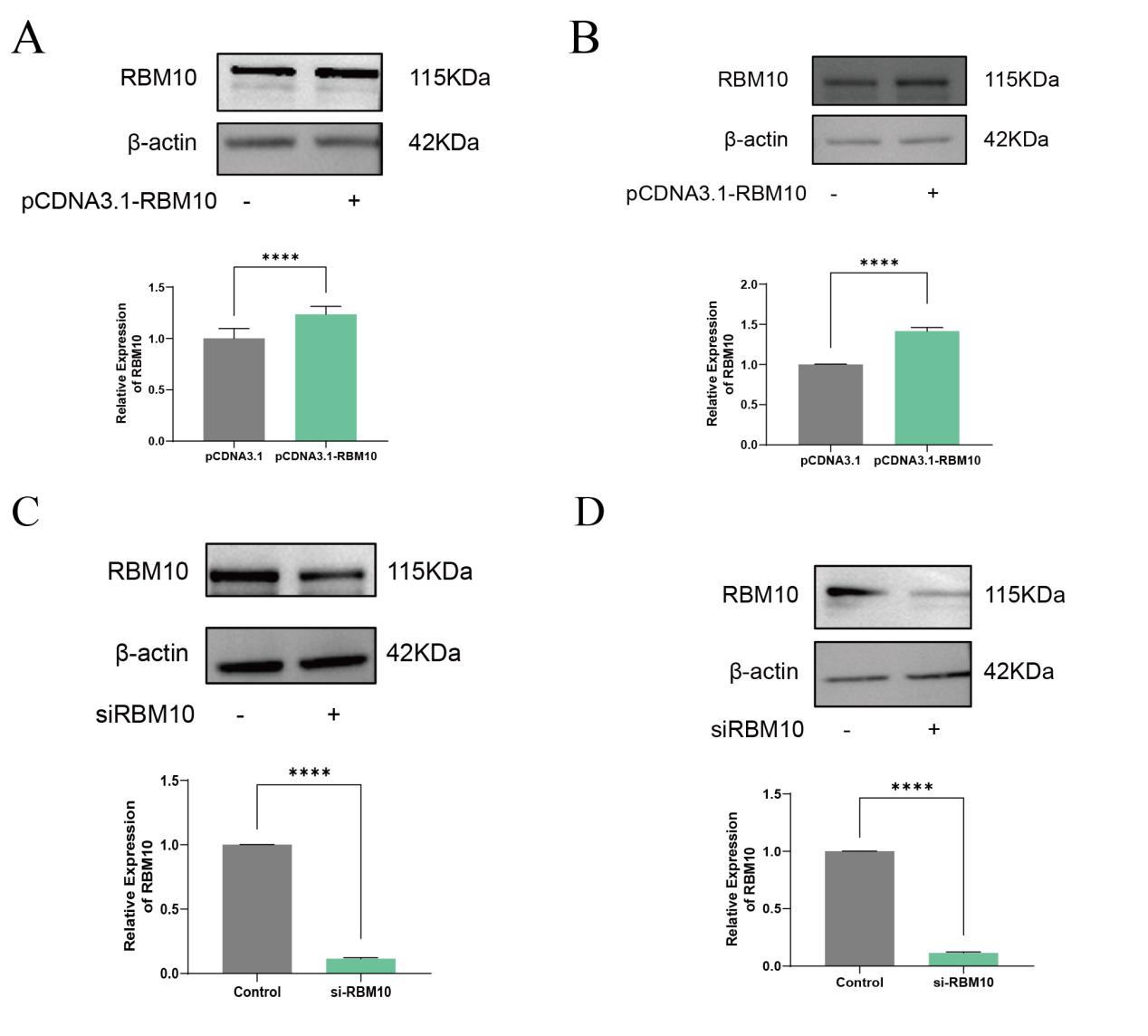


**FIG S6** (A-B) Replicate results of Fig 4B. TZM-bl cells were transfected with the RBM10 overexpression vector for 48 h. The western blot and qPCR were performed to confirm the overexpression of RBM10. (C-D) Replicate results of Fig 4D. TZM-bl cells were transfected with the RBM10 siRNA for 48 h. The western blot and qPCR were performed to validate the knocking down of RBM10 in TZM-bl cells. **** p < 0.0001 vs. the control group.


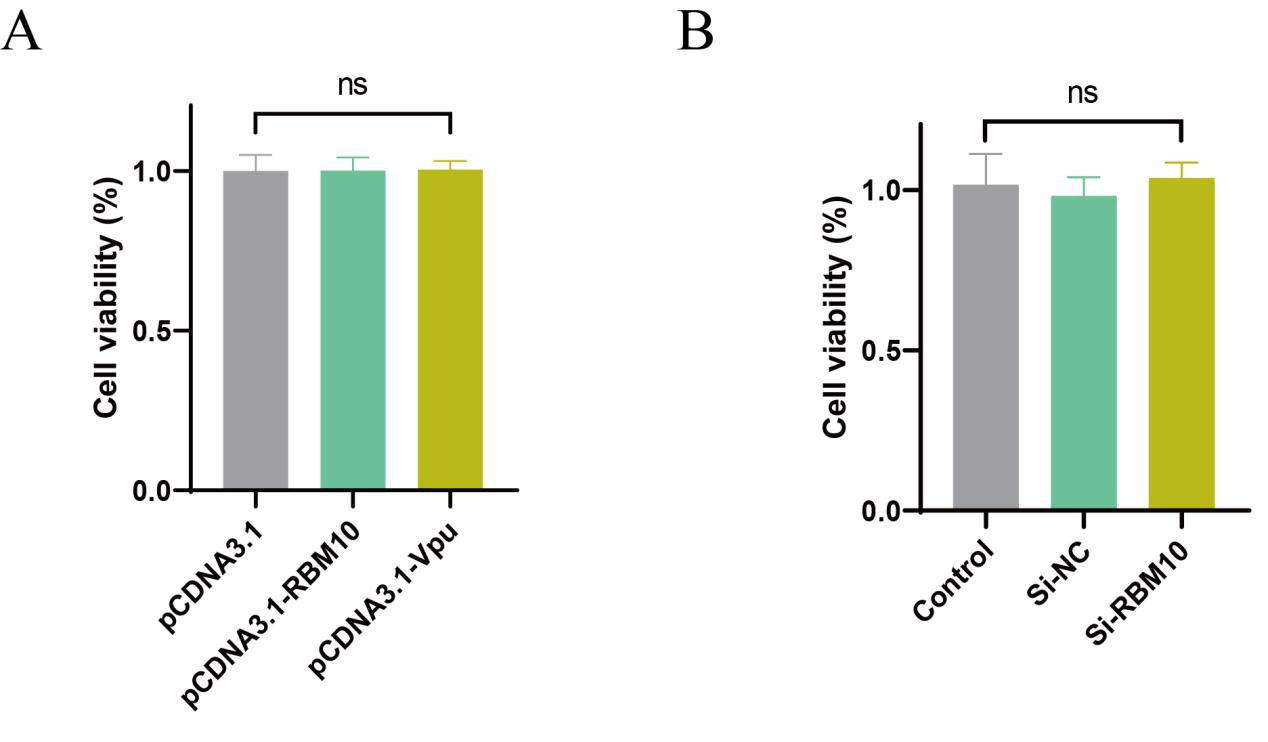


**FIG S7** (A) TZM-bl cells were transfected with RBM10 overexpression vector, Vpu overexpression vector, or control vector. Two days later cell viability was detected by CCK8 assay. (B) TZM-bl cells were transfected with RBM10 knockdown siRNA or negative control siRNA. Two days later cell viability was detected by CCK8 assay.


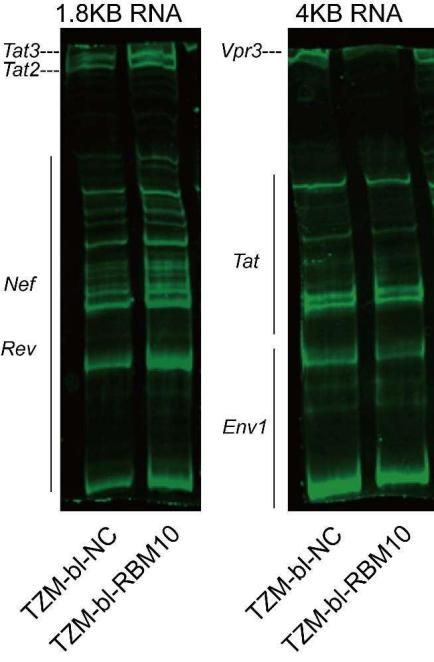


**FIG S8** TZM-bl cells were transfected with RBM10 overexpression vector or control vector, followed by infection with HIV NL4-3 virus for 5 days. FITC-labeled primers that specifically amplify the 1.8 kb and 4 kb HIV-1 RNAs were used for PCR. PCR products were visualized by 6% denaturing polyacrylamide gel electrophoresis.


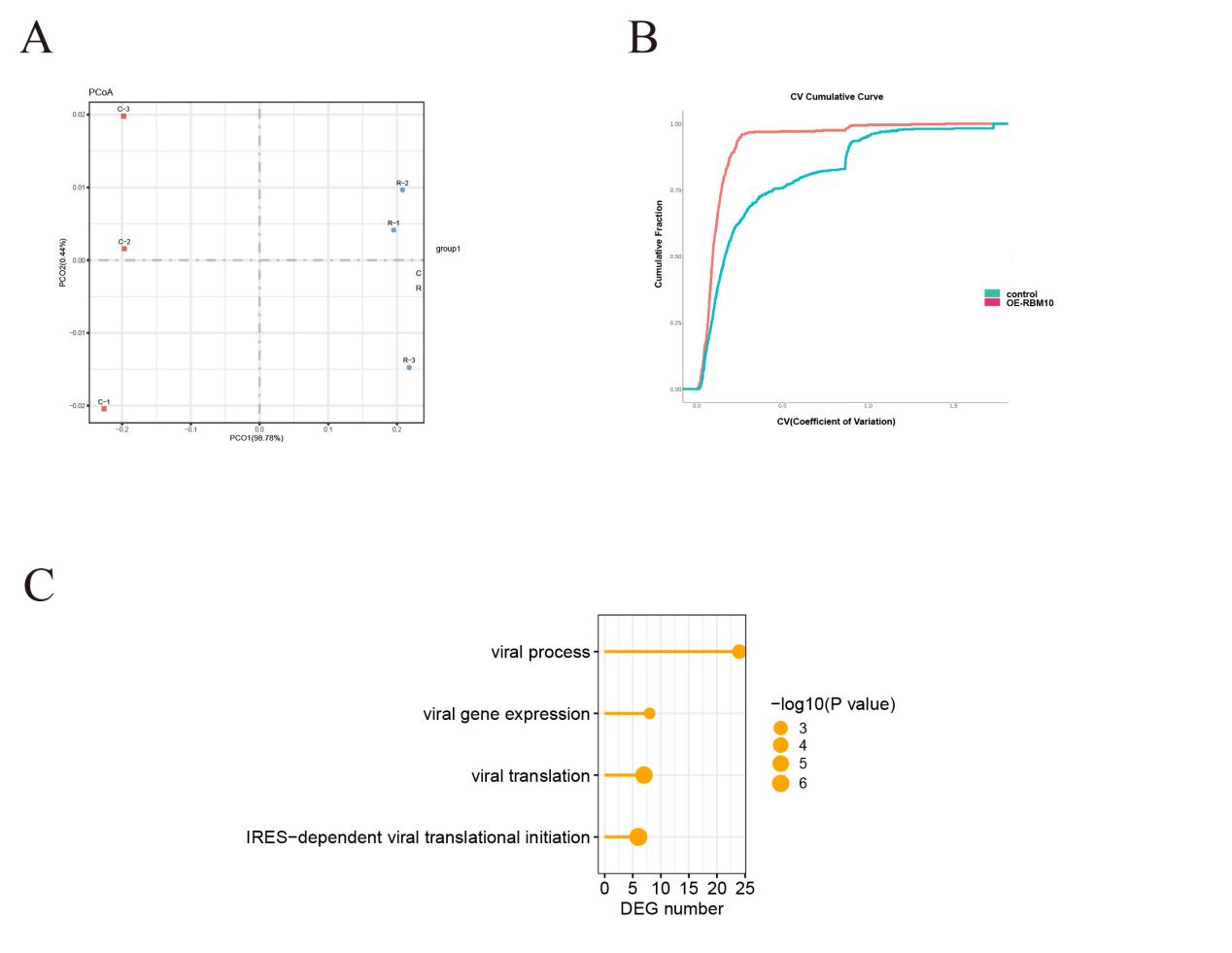


**FIG S9** TZM-bl cells were transfected with RBM10 overexpression or control vectors, followed by infection with the HIV-1 NL4-3 virus. After five days, the proteins were extracted from cells and subjected to proteomic analysis. (**A**) PCA analyses were conducted to determine the differences between groups. (**B**) The CV curve of RBM10 overexpression proteomics. (**C**) GO enrichment analyses were performed to identify significantly enriched GO terms.
